# Macrophage secretory IL-1**β** promotes docetaxel resistance in head and neck squamous carcinoma via SOD2/CAT-ICAM1 signaling

**DOI:** 10.1172/jci.insight.157285

**Published:** 2022-12-08

**Authors:** Ching-Yun Hsieh, Ching-Chan Lin, Yu-Wen Huang, Jong-Hang Chen, Yung-An Tsou, Ling-Chu Chang, Chi-Chen Fan, Chen-Yuan Lin, Wei-Chao Chang

**Affiliations:** 1 Division of Hematology and Oncology, Department of Internal Medicine, China Medical University Hospital, China Medical University, Taichung, Taiwan.; 2 Department of Medical Research, MacKay Memorial Hospital, Taipei, Taiwan.; 3 Department of Otolaryngology-Head and Neck Surgery and; 4 Center for Molecular Medicine, China Medical University Hospital, China Medical University, Taichung, Taiwan.; 5 Chinese Medicinal Research and Development Center, China Medical University Hospital, and; 6 Department of Biological Science and Technology, China Medical University, Taichung, Taiwan.; 7 Department of Research and Development, Marker Exploration Corporation, Taipei, Taiwan.; 8 Department of Medical Laboratory Science and Biotechnology, Yuanpei University of Medical Technology, Hsinchu, Taiwan.

**Keywords:** Oncology, Cancer, Drug therapy, Macrophages

## Abstract

Docetaxel (DTX) combined with cisplatin and 5-fluorouracil has been used as induction chemotherapy for head and neck squamous cell carcinoma (HNSCC). However, the development of acquired resistance remains a major obstacle to treatment response. Tumor-associated macrophages are associated with chemotherapeutic resistance. In the present study, increased infiltration of macrophages into the tumor microenvironment (TME) was significantly associated with shorter overall survival and increased resistance to chemotherapeutic drugs, particularly DTX, in patients with HNSCC. Macrophage coculture induced expression of intercellular adhesion molecule 1 (ICAM1), which promotes stemness and the formation of polyploid giant cancer cells, thereby reducing the efficacy of DTX. Both genetic silencing and pharmacological inhibition of ICAM1 sensitized HNSCC to DTX. Macrophage secretion of IL-1β was found to induce tumor expression of ICAM1. IL-1β neutralization and IL-1 receptor blockade reversed DTX resistance induced by macrophage coculture. IL-1β activated superoxide dismutase 2 and inhibited catalase, thereby modulating intracellular levels of ROS and inducing ICAM1 expression. Arsenic trioxide (ATO) reduced macrophage infiltration into the TME and impaired IL-1β secretion by macrophages. The combinatorial use of ATO enhanced the in vivo efficacy of DTX in a mouse model, which may provide a revolutionary approach to overcoming acquired therapeutic resistance in HNSCC.

## Graphical abstract

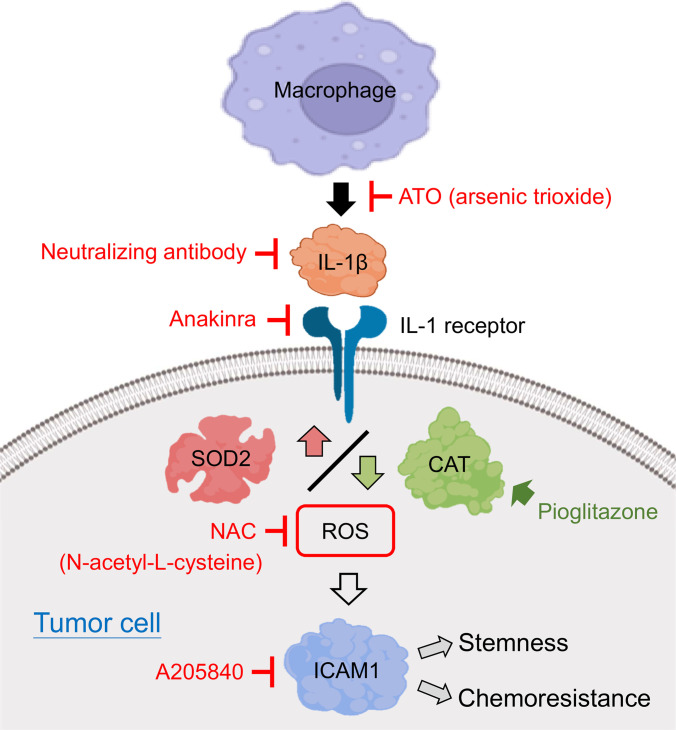

## Introduction

Head and neck squamous cell carcinoma (HNSCC), developing in the outer layer of skin and the mucous membranes of the mouth, nose, and throat, is the sixth most common type of cancer worldwide, accounting for approximately 600,000 new cases annually (1). Early detection of HNSCC is critical to achieve improved outcomes, with reported 5-year survival rates of 70%–90% in patients with stage I and II disease (2). Unfortunately, the majority of patients are diagnosed with advanced-stage disease that is characterized by large tumors accompanied by local invasion and/or distant metastasis, with a reported 5-year overall survival rate of less than 50% in such cases (3). Due to easy accessibility, surgery is the treatment of choice for HNSCC and is associated with high cure rates and reduced morbidity. Chemotherapy combined with radiotherapy is not only considered in patients with unresectable tumors but also serves as an adjuvant therapy in patients with high-risk features (4). Combined treatments include concurrent chemoradiotherapy and induction chemotherapy (ICT), in which cisplatin plus 5-fluorouracil (PF) is the most common regimen (5). Currently, the addition of docetaxel (DTX) to PF ICT (TPF ICT) has been a revolutionary treatment strategy allowing improved patient outcomes and organ preservation (6). Our recent phase II study and previous studies have found that complete response after PF ICT or TPF ICT is significantly associated with greater progression-free survival and overall survival in patients with HNSCC (7–9). However, the development of acquired chemoresistance remains a major obstacle to prolonged treatment response.

Tumor-associated macrophages (TAMs), originating from bone marrow–derived peripheral blood monocytes and tissue-resident macrophages, are the most abundant component of the innate immune system in the tumor microenvironment (TME) (10). Increased infiltration of TAMs has recently been shown to closely correlate with tumor progression and influences therapeutic response and clinical outcomes (11, 12). Cytokines secreted by TAMs, such as TNF-α, IL-6, and IL-10, have been shown to be involved in reciprocal interactions between macrophages and tumor cells leading to increased chemoresistance in both an in vitro macrophage coculture system and an in vivo animal model (13, 14). A crucial role of TAMs in HNSCC progression has been posited in recent studies (15); however, the potential functions of TAMs in HNSCC chemoresistance and the underlying molecular mechanisms have yet to be fully elucidated.

IL-1β, an inflammatory cytokine, is predominantly secreted by activated macrophages and monocytes (16). IL-1β is capable of regulating epithelial-mesenchymal transition (EMT) and is involved in cancer development and distant metastasis (17). IL-1β promotes stemness of tumor cells, supporting the early finding that IL-1β–mediated tumor-stroma interactions confer chemoresistance in pancreatic ductal carcinoma cells through a paracrine effect (18, 19). Recent data from a renal cell carcinoma animal model evaluating the effect of IL-1β on immune checkpoint inhibitor resistance demonstrated the efficacy of blocking IL-1β in promoting tumor regression (20). Thus, IL-1β may serve as a potential therapeutic target for reducing chemoresistance.

In this study, we observed increased TAM infiltration into the TME was significantly associated with poorer chemotherapeutic responses in patients with HNSCC. Multiplex cytokine assays revealed that macrophage-tumor coculture increased macrophage secretion of IL-1β, thereby inducing the expression of intercellular adhesion molecule 1 (ICAM1; also known as CD54) in HNSCC. ICAM1 promoted tumor stemness and increased the resistance of HNSCC to chemotherapy, particularly DTX. Mechanically, IL-1β increased expression of mitochondrial superoxide dismutase 2 (SOD2) and inhibited catalase (CAT) to modulate intracellular levels of reactive oxygen species (ROS), thereby leading to activation of ICAM1. Arsenic trioxide (ATO), a therapeutic agent against acute promyelocytic leukemia (APL), demonstrated an immunomodulatory effect by inhibiting macrophage secretion of IL-1β. Thus, we evaluated the combination of ATO and DTX as a foundation for pharmacological targeting of the IL-1β/ICAM1 axis in a proof-of-concept setting.

## Results

### TAM infiltration is significantly associated with poor chemotherapeutic response in patients with HNSCC.

**Figure 1:**
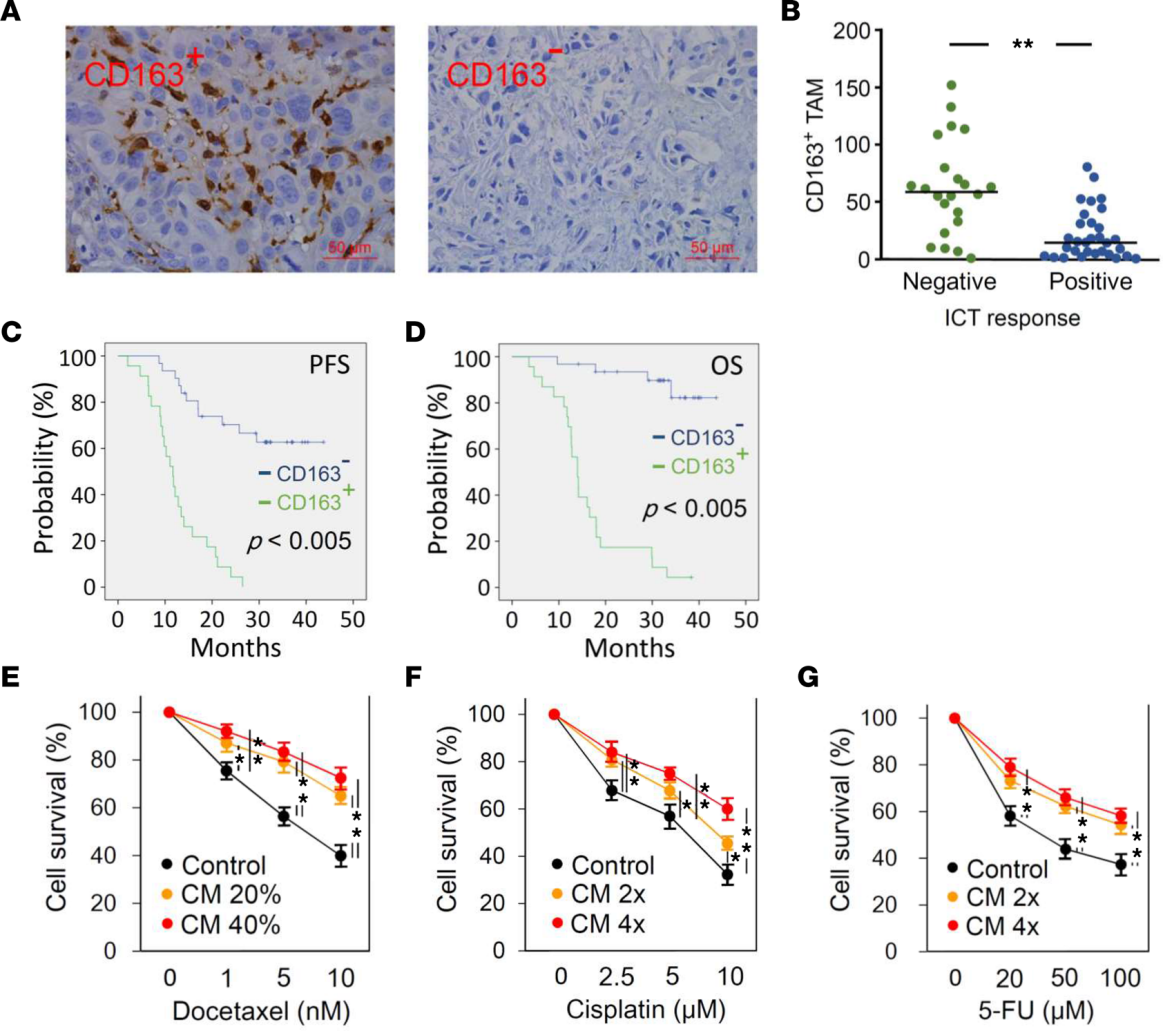
TAMs impair chemotherapeutic response in patients with HNSCC. (**A**) CD163 expression in pathologic specimens of patients with HNSCC was examined by IHC. Representative images demonstrating high CD163 signal (CD163^+^) and low CD163 signal (CD163^–^). Scale bar: 50 μm. (**B**) The correlation between CD163 levels in tissue specimens and therapeutic response to ICT. Positive and negative responses to ICT were defined as ≥70% or <70% decreases in the sum of the longest diameter of the target lesions compared with baseline sum diameters, respectively. Correlations between CD163 levels and (**C**) PFS or (**D**) OS were analyzed using the Kaplan-Meier method. FaDu cells were pretreated with different doses of CM for 24 hours and then treated with indicated doses of (**E**) DTX, (**F**) cisplatin, and (**G**) 5-fluorouracil (5-FU) for a further 48 hours. Cell viability was determined by MTT assay. Data were displayed as the means ± SD. For statistical analyses, a 2-tailed unpaired Student’s *t* test (**B**), log-rank test (**C** and **D**), or 1-way ANOVA with Tukey’s post hoc test (**E**–**G**) was used. *, *P* < 0.05; **, *P* < 0.01.

Compelling evidence indicates that TAMs potentially exert tumor-supporting functions in the TME, thereby limiting therapeutic response (21). To assess the impact of TAMs on chemotherapeutic efficacy, we conducted IHC assays in tissue specimens to determine the correlation between TAM numbers and therapeutic response to ICT in patients with HNSCC. A total of 54 pathologic specimens were collected from patients with HNSCC, with demographic information shown in Table 1. CD163 staining, a marker of macrophages, was used to determine the numbers of TAMs in the TME of tissue specimens. CD163-expressing cells were counted in 4 high-power fields (HPFs) per section. A positive CD163 signal was defined as more than 40 TAMs per HPF, whereas a negative CD163 signal was defined as 40 TAMs or fewer per HPF (Figure 1A). In this study, we defined positive and negative responses to ICT therapy as ≥70% or <70% decreases in the sum of the longest diameter of target lesions compared with baseline, respectively, according to the Response Evaluation Criteria in Solid Tumors (RECIST) 1.1 (22). Statistical analyses demonstrated a significant correlation between CD163^+^ and negative response to ICT in patients with HNSCC (Figure 1B). In addition, clinical analysis demonstrated that CD163^+^ was significantly associated with shorter progression-free survival (PFS) and overall survival (OS) in the present study cohort (Figure 1, C and D).

To determine the impact of macrophages on the efficacy of chemotherapeutic agents against tumor cells, macrophage-like cells differentiated from THP-1 by PMA were cocultured with HNSCC cells for 48 hours. Macrophage-tumor coculture media (CM) were used to treat HNSCC to mimic the interaction of macrophages and tumor cells in the TME. CM treatment significantly enhanced resistance of FaDu and OECM-1 to DTX in a dose-dependent manner (Figure 1E and Supplemental Figure 1; supplemental material available online with this article; https://doi.org/10.1172/jci.insight.157285DS1). Although CM impaired the killing effect of cisplatin and 5-fluorouracil, this effect was only observed at high concentrations of CM (Figure 1, F and G). Accordingly, we aimed to explore the effect of CM on DTX resistance in this study.

### CM-induced ICAM1 increases the stemness of HNSCC.

**Figure 2:**
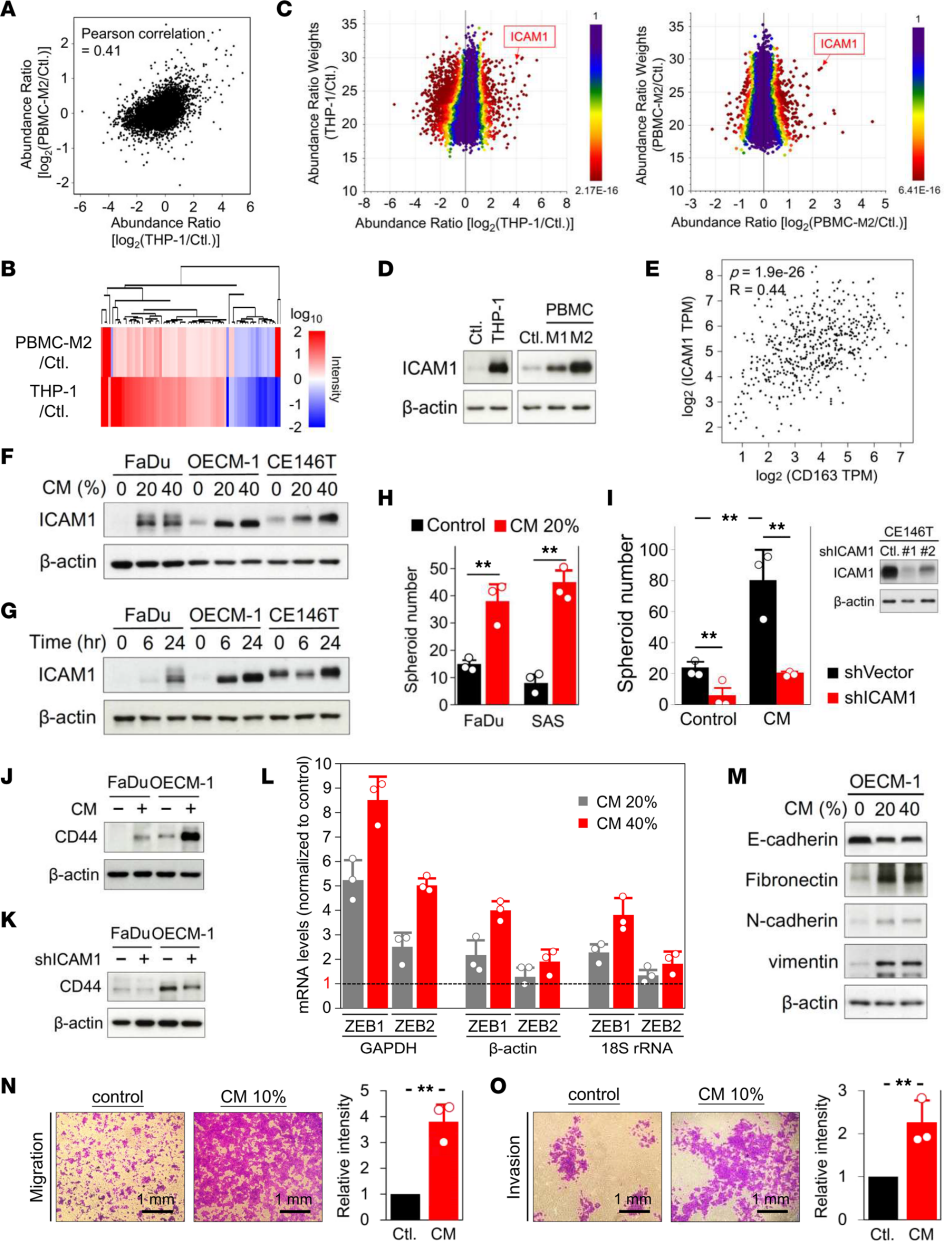
CM-induced ICAM1 increases tumor stemness in HNSCC. (**A**) Pearson’s correlation between commonly identified proteins in both comparative proteomic analyses analyzed using log_2_ ratio of abundance changes. (**B**) Heatmap clustering analysis of quantified proteins with significant abundance ratios (*P* < 0.05) in both comparative proteomes. (**C**) Scatterplots demonstrating the relationship between ratio weights and abundance ratios for each identified protein in the proteomic analysis. The color of protein dots represents the *P* value for corresponding abundance ratios. (**D**) ICAM1 expression in OECM-1 cells cocultured with THP-1–derived macrophages and PBMC M1-like or M2-like macrophages. (**E**) Spearman’s monotonic correlation between ICAM1 and CD163 expression in HNSCC analyzed using the TCGA RNA-Seq database on the GEPIA server. TPM, transcripts per million. ICAM1 in FaDu, OECM-1, and CE146 cells induced by (**F**) CM or (**G**) 20% CM at different periods. (**H**) Spheroid formation in FaDu and SAS cells in the presence or absence of 20% CM for 12 days (*n* = 3). (**I**) Number of spheroids formed in ICAM1-KD and control CE146T cells in the presence or absence of 20% CM for 12 days (*n* = 3). The effect of ICAM1 inhibition by shRNA was validated (right). Effects of (**J**) 20% CM treatment and (**K**) ICAM1-KD on CD44 expression in FaDu and OECM-1 cells. (**L**) mRNA levels of ZEB1 and ZEB2 in OECM-1 cells treated with CM determined by quantitative PCR and normalized to untreated control (set as 1, dashed line). GAPDH, β-actin, and 18S rRNA mRNA were internal controls for gene expression. (**M**) Levels of EMT-related proteins in OECM-1 cells induced by CM. β-actin, loading control. (**N**) Transwell migration and (**O**) invasion assays performed using OECM-1 cells in the presence or absence of 10% CM. Signal quantification with crystal violet extract measured by colorimetric analysis at 570 nm (*n* = 3). Means ± SD. Two-tailed unpaired Student’s *t* test (**H**, **I**, **N**, and **O**). **, *P* < 0.01.

To identify the molecules responsible for CM-induced chemoresistance, we performed quantitative proteomic analyses to explore the alteration of protein signatures in HNSCC cells following treatment of CM. Additionally, to evaluate whether CM derived from THP-1–differentiated macrophages and tumor coculture has utility mimicking communication between TAMs and tumor cells, we simultaneously determined the proteomic changes of HNSCC cells cocultured with CM acquired from PBMC-derived M2-like macrophages (characterized in Supplemental Figure 2), which are currently thought to be an alternative activation phenotype of TAMs. Proteomic results identified 6,821 and 6,774 proteins in the THP-1 and PBMC-M2 groups, respectively (Supplemental Tables 1 and 2). Of note, 6,124 proteins were commonly identified in both groups in an overlapping comparison. Pearson’s correlation coefficient between the abundance changes (log_2_ ratio) of 6,124 proteins in both groups was 0.41, reflecting a positive correlation between whole proteome alternations in response to both CM (Figure 2A). We next performed cluster analysis using quantified proteins with significant abundance ratios (adjusted *P* < 0.05) determined by background *t* test using the computational platform Proteome Discovery (v2.4). Heatmap clustering of significantly changed proteins demonstrated that 93% of proteins (67 out of 72) were consistently up- or downregulated in response to both CM (Figure 2B), indicating CM from THP-1–tumor coculture has utility in evaluating the interaction between TAMs and tumor cells. Proteomic alterations in OECM-1 cells in response to individual CM treatments were visualized on 3-dimensional scatterplots, which revealed the relationship between ratio weights (weighting by mass intensity) and abundance ratios of each protein. The color of protein dots represents adjusted *P* values for abundance ratios from the background *t* test (Figure 2C). Notably, ICAM1 was highly upregulated in OECM-1 cells in response to both CM (Figure 2C). This finding was replicated in CM-treated FaDu cells (Supplemental Figure 3). In line with the proteomic data, ICAM1 induction was confirmed by Western blotting in both coculture systems (Figure 2D). Additionally, M2-like macrophages had higher efficacy in inducing ICAM1 than M1-like macrophages (Figure 2D). A significantly positive correlation between ICAM1 and CD163 expression in patients with HNSCC was found by in silica gene expression analysis using The Cancer Genome Atlas (TCGA) RNA-Seq database (23) (Figure 2E). Furthermore, CM coculture induced tumor-expressed ICAM1 in dose- and time-dependent manners (Figure 2, F and G). The dramatic upregulation of ICAM1 implies a potential role in mediating the interaction between macrophages and tumor cells responsible for chemoresistance in HNSCC.

ICAM1, a cell surface glycoprotein expressed on leukocytes and endothelial cells, is involved in a range of important processes, including cell signaling, cell-cell interactions, and tissue stability (24). ICAM1 can be upregulated by LPS and inflammatory cytokines, such as IFN-γ and TNF-α (25). The oncogenic role of ICAM1 in promoting tumor stemness has been identified in lung cancer, hepatocellular carcinoma, and esophageal squamous cell carcinoma (26–28). Cancer stem cells (CSCs) control the dynamics of tumorigenesis and are generally considered to play critical roles in chemoresistance and tumor metastasis (29). To evaluate whether CM increases the stemness of HNSCC through ICAM1 activation, we determined the impacts of CM on alterations in CSC features. CM treatment significantly increased spheroid formation in HNSCC cells (Figure 2H). In ICAM1^hi^-expressing CE146T, ICAM1 knockdown (ICAM1-KD; Supplemental Table 3) significantly inhibited CM-induced spheroid formation (Figure 2I). CD44 has been recognized as a CSC marker in many types of cancers, including HNSCC (30). We found that CM treatment markedly upregulated the expression of CD44 (Figure 2J), whereas ICAM1-KD suppressed CD44 levels in FaDu and OECM-1 cells (Figure 2K). Additionally, the ICAM1 inhibitor, A205804, attenuated CM-induced CD44 in FaDu cells (Supplemental Figure 4). These results indicate that CM enhanced CSC properties in an ICAM1-dependent manner. Activation of EMT is an important feature of CSCs. EMT is tightly regulated by transcription factors that alter gene expression to promote phenotype alterations leading to functional changes in cell migration and invasion (31). Indeed, the EMT-related transcriptional factors, zinc finger E-box-binding homeobox 1 (ZEB1) and ZEB2, were upregulated in response to CM treatment (Figure 2L). In addition, CM increased expression of the mesenchymal molecule markers, fibronectin, N-cadherin, and vimentin, but suppressed the epithelial marker E-cadherin (Figure 2M). Moreover, CM promoted cell migration and invasion (Figure 2, N and O, and Supplemental Figure 5, A and B). Taken together, these findings indicate that macrophage coculture can increase the stemness of HNSCC through ICAM1 activation and may consequently impair the efficacy of chemotherapy.

### CM-induced ICAM1 enhances HNSCC resistance to DTX.

**Figure 3:**
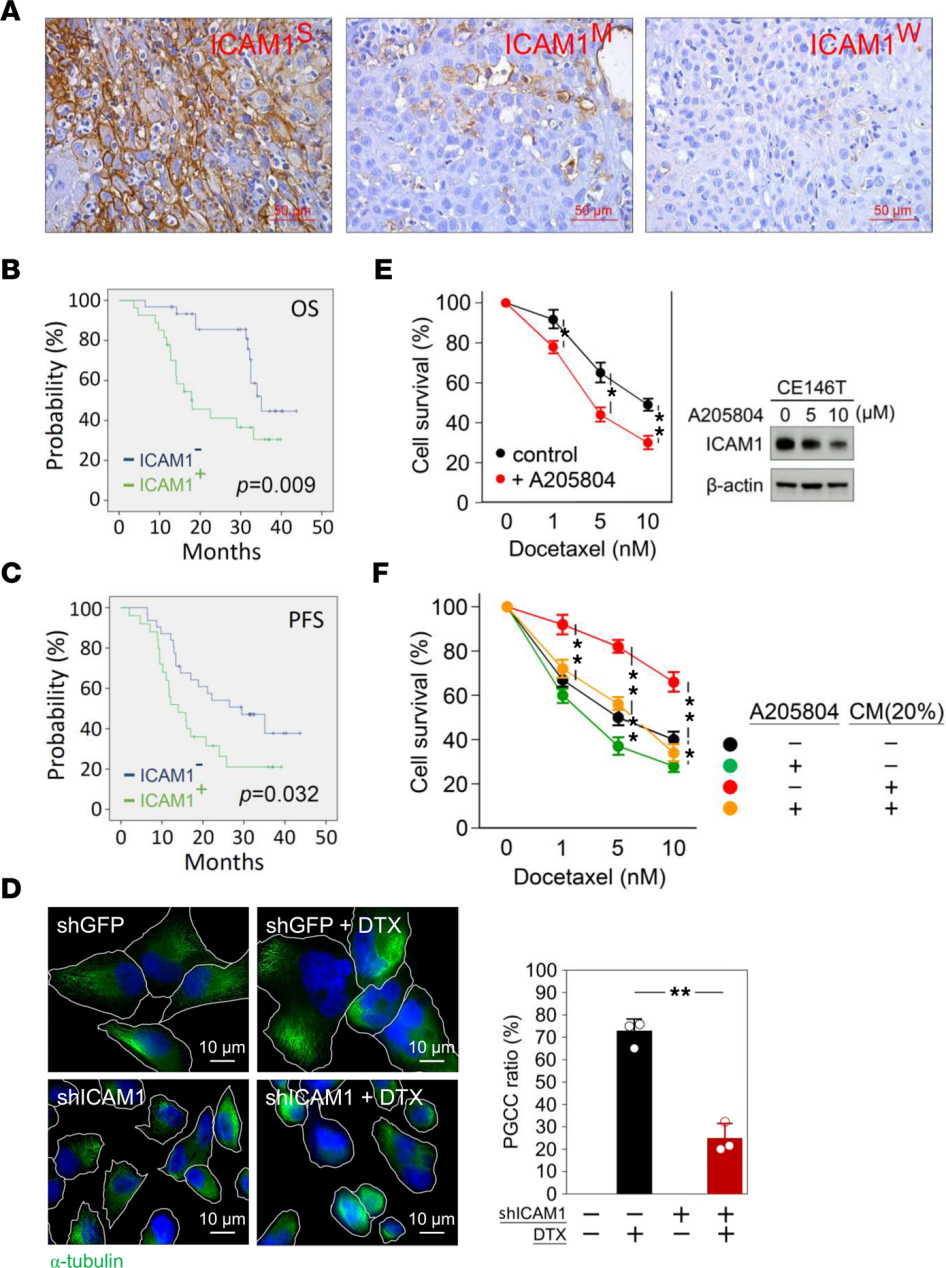
CM-induced ICAM1 enhances HNSCC resistance to DTX. (**A**) Representative IHC images demonstrating strong ICAM1 signal (ICAM1^S^), moderate signal (ICAM1^M^), and weak signal (ICAM1^W^) in pathologic specimens of patients with HNSCC. Correlations between ICAM1 expression and (**B**) OS or (**C**) PFS were analyzed according to ICAM1 signals using the Kaplan-Meier method. (**D**) The expression of α-tubulin in ICAM1-KD and control CE146T cells with or without 10 nM DTX treatment was determined by fluorescence microscopic analysis. DAPI, nuclear staining. (**E**) Viability of CE146T cells with or without treatment with the ICAM1 inhibitor, A205804 (10 μM), with indicated doses of DTX was determined by MTT assay. The effect of A205804 on ICAM1 inhibition was determined by Western blotting (right panel). (**F**) Cell viability of ICAM1-KD and control CE146T cells cultured in indicated doses of DTX in the presence or absence of 20% CM was determined by MTT assay. Data were displayed as the means ± SD. For statistical analyses, a 2-tailed unpaired Student’s *t* test (**E**), log-rank test (**B** and **C**), or 1-way ANOVA with Tukey’s post hoc test (**D** and **F**) was used. *, *P* < 0.05; **, *P* < 0.01.

To examine whether ICAM1 confers DTX resistance on HNSCC, ICAM1 levels in patient specimens were determined by IHC analyses. The results demonstrated differential expression of ICAM1 in our patient cohort, defined as strong signal (ICAM1^S^), moderate signal (ICAM1^M^), and weak signal (ICAM1^W^) (Figure 3A). Tissue specimens with ICAM1^S^ were classified as ICAM1^+^, while those with ICAM1^M^ and ICAM1^W^ were classified as ICAM1^–^. Kaplan-Meier survival analysis revealed a significant correlation between higher ICAM1 protein levels and shorter OS and PFS in patients with HNSCC (Figure 3, B and C). DTX stabilizes the structure of microtubules as an inhibitor of cellular mitosis and potentially induces the formation of polyploid giant cancer cells (PGCCs), which have been shown to enhance stemness and EMT as well as driving resistance to DTX chemotherapy (32–34). Our fluorescence microscopic analysis revealed large numbers of PGCCs in ICAM1^hi^-expressing CE146T within 24 hours of DTX treatment, whereas ICAM1-KD dramatically reduced DTX-induced PGCC formation (Figure 3D), implicating ICAM1 involvement in DTX-induced PGCC formation by promoting resistance to DTX. Indeed, treatment with the ICAM1 inhibitor, A205804, sensitized tumor cells to DTX (Figure 3E). Moreover, ICAM1-KD significantly attenuated CM treatment–related DTX resistance, indicating CM induces DTX resistance in an ICAM1-dependent manner (Figure 3F).

### CM modulates intracellular ROS to promote ICAM1 expression.

**Figure 4:**
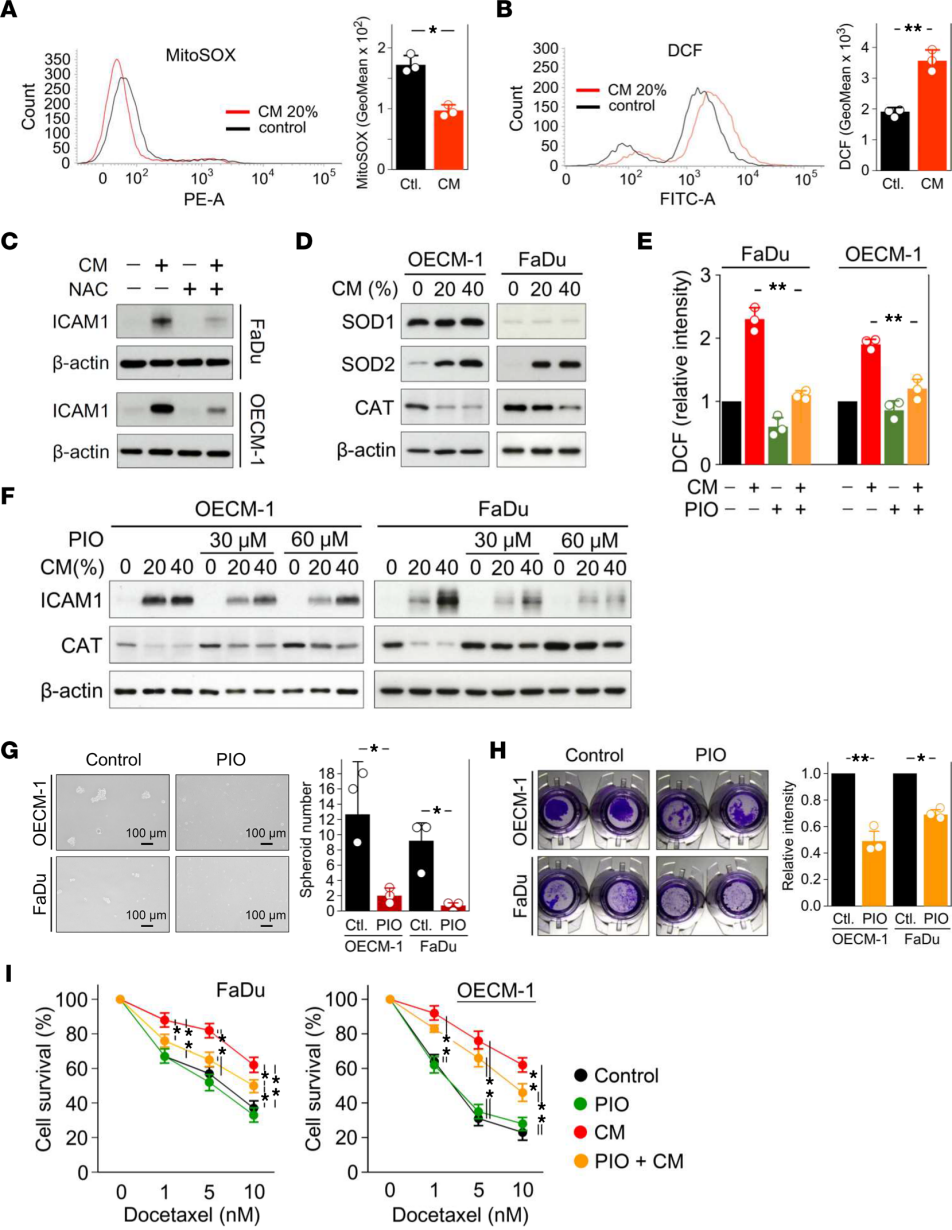
CM enhances ICAM1 expression through modulation of intracellular ROS levels. (**A**) Mitochondrial superoxide levels and (**B**) intracellular ROS levels in FaDu cells with or without 20% CM treatment were determined using the tracer dyes, MitoSOX (Invitrogen) and CM-H2DCFDA (DCF, Invitrogen), respectively. (**C**) The expression of ICAM1 in FaDu and OECM-1 cells with or without 5 mM NAC pretreatment in the presence or absence of 20% CM for 24 hours was determined by Western blotting. (**D**) The expression levels of SOD1, SOD2, and CAT in FaDu and OECM-1 cells treated with indicated doses of CM were determined by Western blotting. (**E**) Intracellular ROS levels in FaDu and OECM-1 cells in the presence or absence of 20% CM or 30 μM PIO were determined using the tracer dye, DCF, by flow cytometry. (**F**) The protein levels of ICAM1 and CAT in FaDu and OECM-1 cells in the presence or absence of indicated doses of CM or 30 or 60 μM PIO were determined by Western blotting. β-actin, loading control. (**G**) Spheroid formation and (**H**) cell invasion of FaDu and OECM-1 cells with or without 30 μM PIO treatment. (**I**) Cell viability of FaDu and OECM-1 cells in the presence or absence of 20% CM or 30 μM PIO for 24 hours was determined under indicated doses of DTX by MTT assay. Data were displayed as the means ± SD. For statistical analyses, a 2-tailed unpaired Student’s *t* test (**A**, **B**, **G**, and **H**) or 1-way ANOVA with Tukey’s post hoc test (**E** and **I**) was used. *, *P* < 0.05; **, *P* < 0.01.

Notably, our proteomic analyses revealed upregulation of SOD2 in both OECM-1 and FaDu cells in response to CM treatment (Supplemental Tables 1 and 2 and Supplemental Figure 3), implying the potential ability of CM to modulate redox homeostasis. Elevated ROS levels are recognized as a distinct characteristic of drug resistance in cancer (35); we therefore determined the effect of CM on regulating intracellular ROS levels. Mitochondrial superoxide and intracellular ROS levels were determined by flow cytometry using MitoSOX (Invitrogen) and CM-H2DCFDA (DCF, Invitrogen) tracer dyes, respectively. Despite reductions in mitochondrial superoxide levels (Figure 4A), CM increased intracellular ROS levels (Figure 4B). To evaluate the correlation between increased intracellular ROS levels and DTX resistance, we determined whether intracellular ROS levels are associated with CM-induced ICAM1 expression. The ROS scavenger, *N*-acetylcysteine (NAC), attenuated CM-induced ICAM1 expression (Figure 4C), indicating that CM promote ICAM1 expression via activation of ROS-related signaling pathways. To further understand the key factors involved in ICAM1 activation, we examined ROS generation and elimination systems, including NADPH oxidases (NOXs) and glutathione peroxidases (GPxs), which may enhance chemoresistance (36, 37). The expression levels of NOXs and GPx1/2 and 4 were unaffected by CM (Supplemental Figure 6, A and B). CM increased expression of the upstream ROS regulator, SOD2, which converts anion superoxide into hydrogen peroxide, but reduced expression of CAT, which converts hydrogen peroxide into water (Figure 4D), thereby leading to increased levels of intracellular ROS. The phenomenon of increased SOD2 levels and decreased CAT levels has previously been observed in tumor cells from advanced stages of disease and has been shown to promote tumor cell proliferation and invasive and migratory phenotypes (38). To determine whether CM-induced ICAM1 is mediated by CAT inhibition, we examined the effect of pioglitazone (PIO), a pharmaceutic thiazolidinedione insulin sensitizer and known CAT agonist, on ICAM1 induction in response to CM treatment. As expected, PIO significantly attenuated the increase in intracellular ROS levels in CM-treated HNSCC cells (Figure 4E) and CM-induced ICAM1 protein levels on Western blotting (Figure 4F). Of note, the effect of PIO on ICAM1 inhibition was inversely correlated with the concentration of CM (Figure 4F). PIO-mediated inhibition of ICAM1 potentially contributed to reduced spheroid formation (Figure 4G) and cell invasion (Figure 4H) as well as sensitization to DTX in CM-treated HNSCC cells (Figure 4I). Collectively, these results demonstrate that CM modulates intracellular ROS generation and regulates ICAM1 expression through inhibition of CAT.

### Macrophage secretory IL-1β induces ICAM1 in HNSCC.

**Figure 5:**
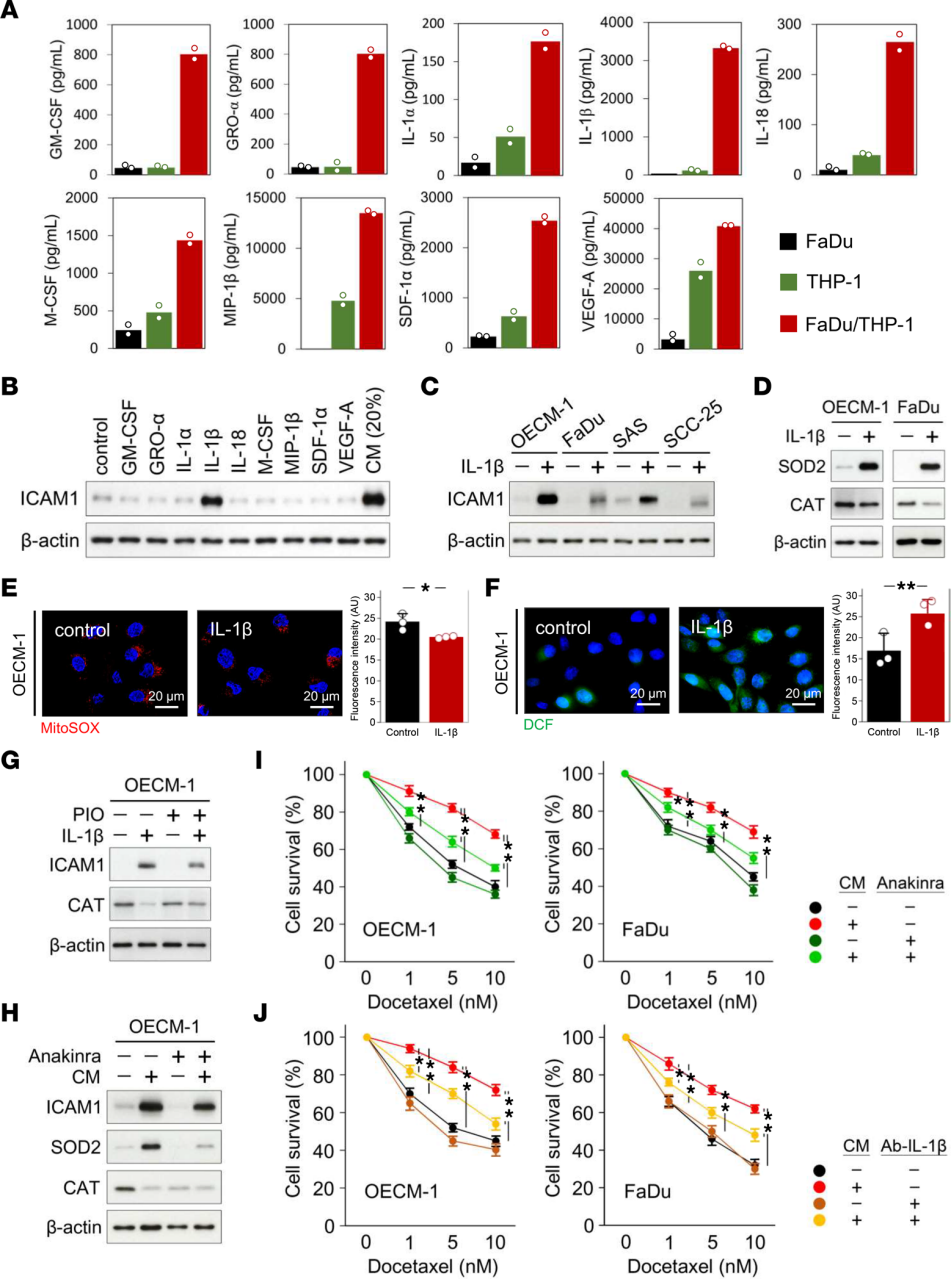
Macrophage secretory IL-1β induces ICAM1 in HNSCC. (**A**) Cytokine levels in CM from monocultures of FaDu or THP-1, or FaDu–THP-1 coculture, were analyzed by Luminex Multi-Analyte Profiling (xMAP) system. (**B**) Expression levels of ICAM1 in OECM-1 cells induced by various kinds of cytokines were determined by Western blotting. (**C**) Expression levels of ICAM1 in different HNSCC cell lines induced by 3 ng/mL IL-1β were determined by Western blotting. (**D**) Expression levels of SOD and CAT in FaDu and OECM-1 cells induced by 3 ng/mL IL-1β were determined by Western blotting. Mitochondrial superoxide levels and (**B**) intracellular ROS levels in OECM-1 cells in the presence or absence of 3 ng/mL IL-1β were monitored using the tracer dyes, (**E**) MitoSOX and (**F**) DCF. (**G**) Expression levels of ICAM1 and CAT in OECM-1 cells in the presence or absence of 3 ng/mL IL-1β or 30 μM PIO for 24 hours were determined by Western blotting. (**H**) Expression levels of ICAM1, SOD2, and CAT in OECM-1 cells in the presence or absence of 20% CM or 50 nM anakinra for 24 hours were determined by Western blotting. β-actin, loading control. Viability of OECM-1 cells with or without 20% CM treatment in the presence or absence of (**I**) 50 nM anakinra or (**J**) 3 ng/mL IL-1β neutralizing antibody (4H5; InvivoGen) was determined under indicated doses of DTX by MTT assay. Data were displayed as the means ± SD. For statistical analyses, a 2-tailed unpaired Student’s *t* test (**E** and **F**) or 1-way ANOVA with Tukey’s post hoc test (**I** and **J**) was used. *, *P* < 0.05; **, *P* < 0.01.

Accumulating evidence suggests that cytokines secreted by TAMs play an important role in chemoresistance (13, 14). To determine the key cytokines secreted by macrophages involved in the induction of ICAM1 in HNSCC, we measured cytokine levels in CM from a tumor–THP-1 coculture system. A total of 65 cytokines were quantitatively measured using the Human ProcartaPlex Immune Monitoring Panel (Invitrogen) by Luminex Multi-Analyte Profiling (xMAP) technology (Supplemental Table 4). Compared with monoculture of FaDu or THP-1 cells, FaDu–THP-1 coculture markedly increased the levels of 9 cytokines in CM, including granulocyte-macrophage colony-stimulating factor (GM-CSF), growth-regulated oncogene-α (GRO-α), IL-1α, IL-1β, IL-18, M-CSF, macrophage inflammatory protein 1β (MIP-1β), stromal cell-derived factor 1α (SDF-1α), and VEGF-A (Figure 5A). To determine the key cytokines responsible for the induction of ICAM1 expression in HNSCC, individual cytokines were used to stimulate OECM-1 cells. Western blotting demonstrated the dominant role of IL-1β in ICAM1 induction in OECM-1 cells (Figure 5B). Additionally, IL-1β induced ICAM1 in other HNSCC cells, including FaDu, SAS, and SCC-25 cells (Figure 5C). Consistent with our previous findings, IL-1β enhanced SOD2 but inhibited CAT in HNSCC cells (Figure 5D). The morphological effects of reduced superoxide and increased intracellular ROS levels were observed by fluorescence microscopic analyses using MitoSOX and DCF tracer dyes, respectively (Figure 5, E and F). The CAT agonist, PIO, reduced IL-1β–induced ICAM1 expression (Figure 5G), suggesting that IL-1β regulates ICAM1 via modulating CAT-related ROS signaling. In addition, the IL-1 receptor (IL-1R) antagonist, anakinra (39), attenuated CM-induced ICAM1 and SOD2 expression (Figure 5H). However, the rescue effect of anakinra on CAT levels was unapparent, which may be due to excessive treatment duration or the properties of the target cell (Figure 5H). We next used anakinra and a neutralizing antibody against human IL-1β (InvivoGen) to determine the causal relationship between CM and IL-1β in DTX resistance. IL-1β treatment enhanced resistance of FaDu and OECM-1 cells to DTX (Supplemental Figure 7, A and B), whereas anakinra sensitized CM-treated HNSCC cells to DTX (Figure 5I). As IL-1R mediates the binding of IL-1β to activate downstream signaling, this result demonstrates an essential role of IL-1β in CM-induced DTX resistance. On the other hand, pretreatment with the IL-1β neutralizing antibody was also able to reverse CM-caused DTX resistance (Figure 5J). Taken together, these data suggest that IL-1β is the major cytokine in CM responsible for ICAM1 induction and DTX resistance in HNSCC.

### ATO reduces IL-1β secretion from macrophages.

**Figure 6:**
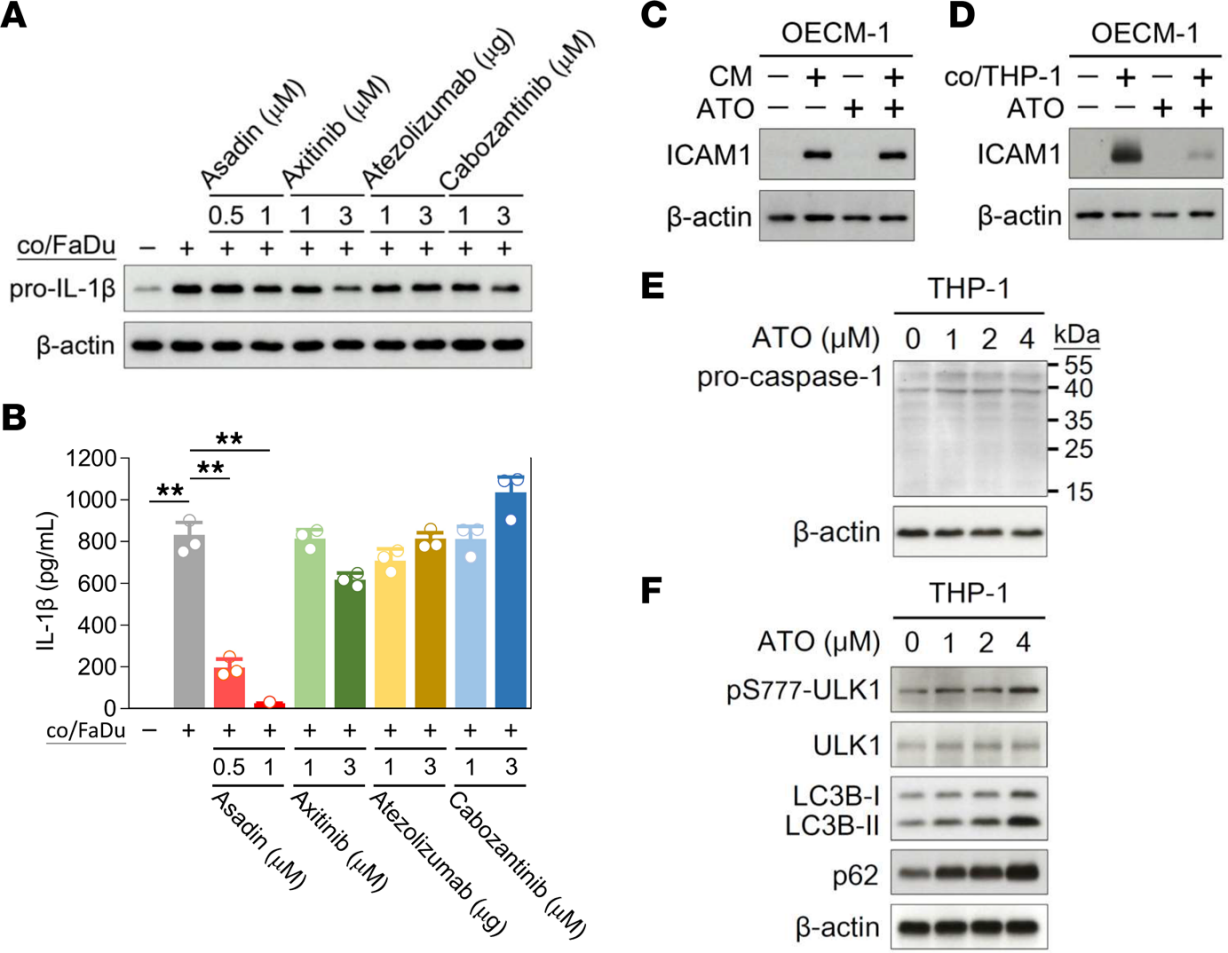
ATO reduces IL-1β secretion from macrophages. THP-1–differentiated macrophages cocultured with FaDu cells were treated with indicated dose of chemotherapeutic agents including Asadin (ATO), axitinib, atezolizumab, and cabozantinib. (**A**) Expression levels of pro–IL-1β in macrophages were determined by Western blotting. (**B**) Levels of mature IL-1β in CM were measured using ELISA kits (ARG80101; Arigo Biolaboratories). (**C**) Expression levels of ICAM1 in OECM-1 cells with or without 20% CM treatment in the presence or absence of 1 μM ATO for 24 hours were determined by Western blot assay. (**D**) Expression levels of ICAM1 in OECM-1 cells with or without coculture with THP-1–differentiated macrophages in the presence or absence of 1 μM ATO for 24 hours were determined by Western blotting. Expression levels of (**E**) caspase-1 and (**F**) autophagy-related proteins such as ULK1, LC3B, and p62 in THP-1–differentiated macrophages treated with indicated doses of ATO were determined by Western blotting. β-Actin, loading control. Data were displayed as the means ± SD. For statistical analysis, 1-way ANOVA with Tukey’s post hoc test (**B**) was used. **, *P* < 0.01.

As the extent of macrophage infiltration contributes to tumor progression and therapeutic resistance, emerging strategies have recently been developed to target macrophages within the TME, including macrophage depleting, modifying macrophage recruitment, and macrophage reprogramming (11, 12). Several pharmacological inhibitors against macrophages have shown great promise in mouse models; however, these agents have yet to be approved for clinical practice (40). To reduce macrophage-induced DTX resistance, we tested available clinical antitumor agents that have been identified to have utility in macrophage modulation, including Asadin (ATO) (41), axitinib (42), atezolizumab (43), and cabozantinib (20). Aside from axitinib, which had a small effect, these agents had no effect on levels of proform IL-1β (pro–IL-1β) in THP-1 cells under FaDu coculture conditions (Figure 6A). Surprisingly, ATO significantly reduced IL-1β levels in CM in a dose-dependent manner (Figure 6B). To further determine whether ATO regulates IL-1β secretion by modulating macrophage function, we measured ICAM1 levels in HNSCC cells after combinatorial treatment with ATO and CM or the addition of ATO to the THP-1–tumor cell coculture system. In HNSCC cells, ICAM1 levels were unaltered after combinatorial treatment with ATO and CM compared with CM treatment alone (Figure 6C). However, when ATO was added to the THP-1–tumor cell coculture system, ICAM1 induction was attenuated in HNSCC cells (Figure 6D), suggesting that ATO modulates IL-1β secretion by modulating macrophage function.

The secretion of mature IL-1β is well-known to be regulated by activation of caspase-1 and secretory autophagy. Pro–IL-1β is proteolytically converted into its secretory mature form by caspase-1, with caspase-1 deficiency resulting in defects in the maturation of pro–IL-1β (44). Western blotting demonstrated no differences in pro–caspase-1 levels and the apparent absence of activated caspase-1 (molecular weight, approximately 20 kDa) in THP-1 cells treated with various doses of ATO (Figure 6E). Due to the lack of a secretory signal sequence, IL-1β is secreted by the secretory autophagy pathway, which is an alternative to the classic endoplasmic reticulum–Golgi route (45). Moreover, an unobstructed autophagy pathway is necessary for IL-1β secretion, with inhibition of autophagy flux shown to reduce IL-1β secretion (46). We found that ATO promoted the initiation of autophagy through activation of unc-51 like autophagy activating kinase 1 (ULK1) and increased the microtubule-associated protein 1 light chain 3B (LC3B) II/I ratio, an indicator of autophagic activity (46) (Figure 6F). However, ATO simultaneously resulted in accumulation of sequestosome 1 (SQSTM1; p62), indicating blockade of autophagic flux (47) (Figure 6F). Consequently, ATO may inhibit IL-1β secretion by preventing secretory autophagy in macrophages.

### ATO improves the efficacy of DTX in a mouse model of HNSCC.

**Figure 7:**
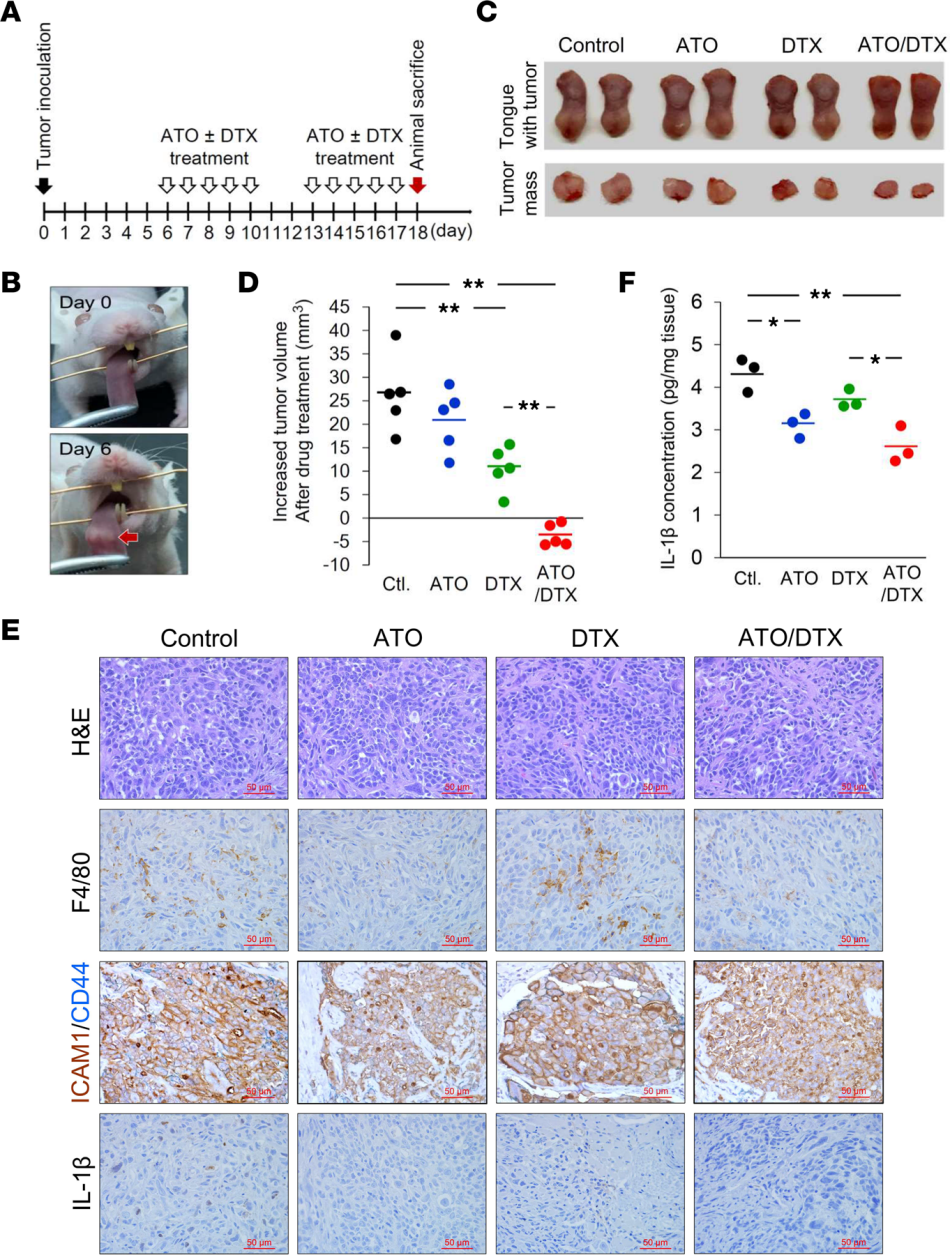
ATO improves the efficacy of DTX in a nude mouse model of HNSCC. (**A**) Schematic illustration of the animal experiment. (**B**) Representative images of the tongue before tumor cell inoculation (day 0) and a visible tumor mass (indicated by arrow) in the tongue (day 6). (**C**) Animals were sacrificed on day 18. Representative images demonstrating tumor masses in the tongues of mice treated with different chemotherapeutic drugs. (**D**) Tumor volume increases in individual mice after drug treatments (from day 6 to day 18) were calculated and plotted. Short bars indicate the average increase in tumor volume for each group. (**E**) Tumor masses were sectioned and embedded in paraffin. IHC analyses were performed with the indicated antibodies. Scale bar, 50 μm. (**F**) Mouse IL-1β concentrations in tumors were measured using ELISA kits (ARG80196; Arigo Biolaboratories). Data were displayed as the means. For statistical analyses, 1-way ANOVA with Tukey’s post hoc test (**D** and **F**) was used. *, *P* < 0.05; **, *P* < 0.01.

To evaluate the efficacy of ATO in sensitizing HNSCC to DTX in vivo, 1 × 10^6^ OECM-1 cells (and SAS cells; results for SAS cells shown in Supplemental Figure 8) were orthotopically inoculated into the tongue of BALB/c nude mice (Figure 7). A schematic of the animal experiment is in Figure 7A. Tumors were clearly visible at 6 days after injection (Figure 7B). Animals were randomly assigned to 4 groups: control, ATO alone, DTX alone, and combination of ATO and DTX. Treatments were administered for 5 consecutive days and 2 days’ rest for 2 cycles. When administered alone, DTX exhibited greater efficacy in reducing tumor growth than ATO, which had almost no effect on tumor growth. The combination of ATO and DTX demonstrated significantly improved efficacy in inhibiting the growth of HNSCC cells (Figure 7, C and D, and Supplemental Figure 8, C and D). Immunohistochemical analysis of FFPE tongue tissues with F4/80, a unique marker of murine macrophages, demonstrated that ATO markedly decreased macrophage infiltration (Figure 7E). In addition, ATO simultaneously attenuated the expression of ICAM1 and its related CSC marker, CD44, in tumor tissues on costaining analysis (Figure 7E). Moreover, ATO reduced IL-1β expression (Figure 7E), with reduced IL-1β levels in tumor masses also observed on ELISA (Figure 7F). These results demonstrate that ATO alone or in combination with DTX can significantly reduce tumoral levels of IL-1β, indicating ATO treatment may modulate IL-1β secretion from TAMs.

**Figure 8:**
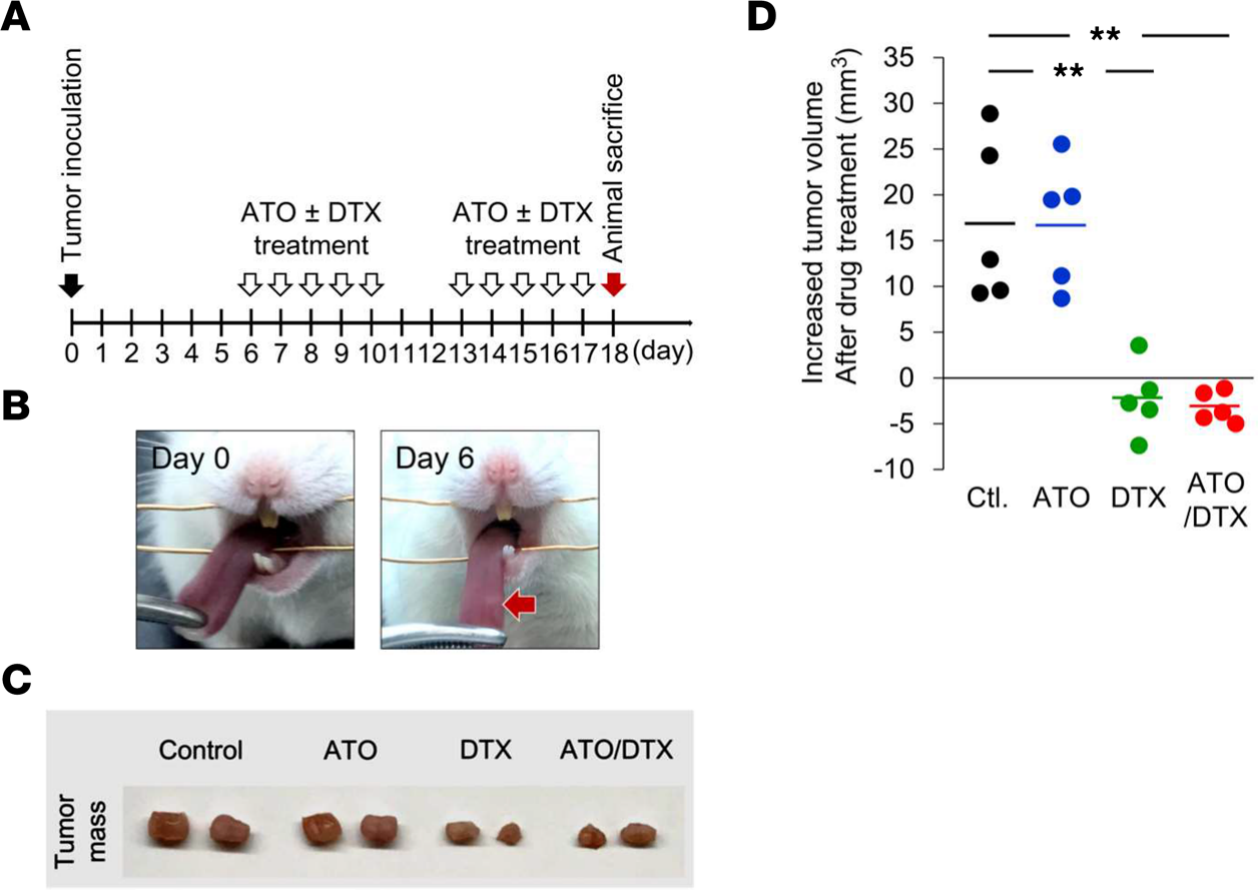
Loss of ATO and DTX synergy in a NOD/SCID mouse model. (**A**) Schematic illustration of the animal experiment. (**B**) Representative images of the tongue before tumor cell inoculation (day 0) and a visible tumor mass (indicated by arrow) in the tongue (day 6). (**C**) Animals were sacrificed on day 18. Representative images demonstrating tumor masses in the tongues of mice treated with different chemotherapeutic drugs. (**D**) Tumor volume increases in individual mice after drug treatments (from day 6 to day 18) were calculated and plotted. Short bars indicate the average increase in tumor volume for each group. Data were displayed as the means. For statistical analysis, 1-way ANOVA with Tukey’s post hoc test (**D**) was used. **, *P* < 0.01.

To further verify the synergistic effect between ATO and DTX combination bypassing macrophage-induced chemoresistance, we repeated the in vivo experiment in macrophage-lacking NOD/SCID mice (Figure 8). DTX had greater efficacy in inhibiting tumor growth in NOD/SCID mice than in nude mice (Figure 8D and Figure 7D); however, no synergistic effect was observed between ATO and DTX in the absence of macrophages (Figure 8D). This result strongly suggests that the synergistic effect of ATO and DTX in nude mice is mediated by modulation of macrophage-induced chemoresistance.

**Figure 9:**
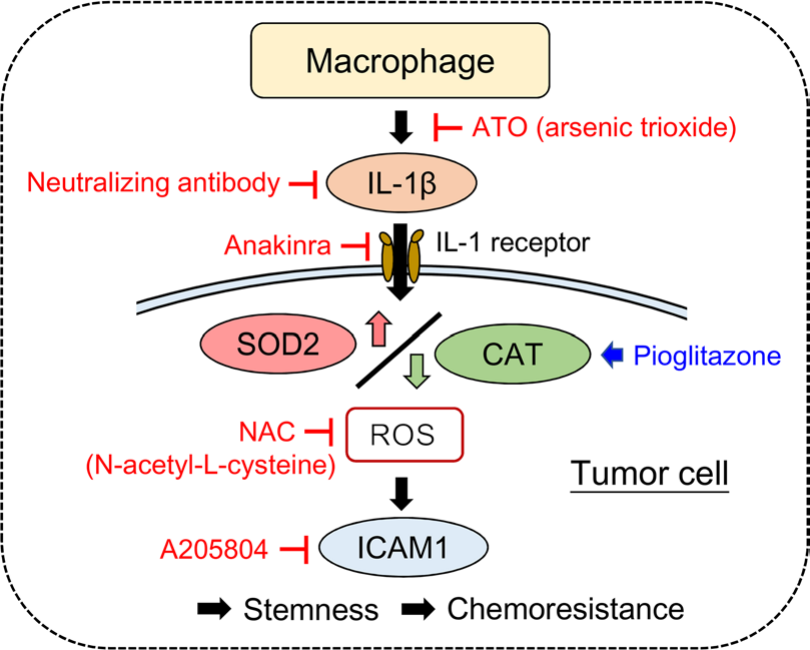
Representative working model. IL-1β secreted by TAMs activates SOD2 and inhibits CAT to modulate intracellular ROS levels, thereby inducing ICAM1 expression. ICAM1 increases tumor stemness and PGCC formation, thereby promoting DTX resistance in HNSCC. Pharmaceutical inhibitors or agents against the IL-1β-SOD2/CAT-ICAM1 pathway may sensitize HNSCC to DTX. The clinical drug, ATO, reduces macrophage infiltration and attenuates IL-1β secretion by targeting macrophages, thereby potentially improving the efficacy of DTX in treating HNSCC.

Collectively, our data demonstrate that TAMs confer DTX resistance in HNSCC via secretion of IL-1β, which activates SOD2 and inhibits CAT to modulate intracellular ROS levels, thereby increasing ICAM1 expression in HNSCC. ICAM1 promotes tumor stemness and PGCC formation, thereby reducing the effects of DTX in HNSCC. ATO potentially reduces infiltration of macrophages into the TME and impairs IL-1β secretion from macrophages, indicating a potential role for ATO in overcoming the chemotherapeutic resistance generated by the interaction between TAMs and HNSCC. Our finding may provide an alternate strategy for improving therapeutic efficacy in HNSCC, with a representative working model summarized in Figure 9.

## Discussion

DTX, an inhibitor of mitosis, is used clinically in the treatment of ovarian, breast, lung, esophageal, prostate, bladder, and head and neck cancers. The development of tumor resistance is the main limitation of DTX therapy. Drug efflux plays a critical role in DTX resistance, with overexpression of ATP-binding cassette sub-family B member 1 and sub-family C member 5 identified to rescue tumor cells from DTX cytotoxicity (48). The inhibition of apoptosis represents another main mechanism of DTX resistance. In resistant HNSCC, activation of NF-κB signaling and overexpression of survivin suppresses activity of caspase-9, leading to inhibition of apoptosis (49). Unfortunately, strategies that target these mechanisms underlying tumor resistance remain under development. We report a mechanism of DTX resistance in HNSCC mediated by the secretion of IL-1β from macrophages to induce ICAM1 expression, which enhances the stemness of tumor cells and promotes PGCC formation in response to DTX treatment, thereby leading to increased chemoresistance. ICAM1 is a surface glycoprotein with cell adhesion activity. Accordingly, its roles in the initiation and progression of tumor metastasis have been intensively studied and are well established (29). Although a close correlation between ICAM1 expression and chemotherapeutic resistance has been described in previous studies (50, 51), the mechanisms underlying ICAM1-driven chemoresistance have yet to be fully elucidated. Different from primary tumor cells, PGCCs with molecular and cellular diversity harbor phenotypic and functional heterogeneity within a complex tumor architecture. PGCCs are posited to have prominent roles in tumorigenesis, metastasis, therapeutic repopulation, and treatment resistance (32, 52). Although digital pathology and artificial intelligence–assisted methods may help identify PGCCs in clinical specimens, a lack of reliable PGCC biomarkers has limited the development of effective identification and elimination strategies (53). The potentially novel function of ICAM1 in promoting PGCC formation may shed light on the biological mechanisms underlying PGCC development and serve as a potential target for PGCC eradication.

IL-1β, a pleiotropic cytokine, has broad effects in hematopoiesis, inflammatory responses, and immune activities. In the TME, IL-1β has protumoral properties in promoting angiogenesis and cancer metastasis in a range of cancer types, including breast cancer, liver cancer, colon cancer, and melanoma (54). Further, IL-1β has been shown to be closely linked with chemoresistance in solid tumors. IL-1β induces upregulation of baculoviral IAP repeat containing 3, which is involved in resistance to doxorubicin in breast cancer (55). Mononuclear cell–derived IL-1β confers camptothecin resistance in pancreatic cancer by upregulating cyclooxygenase-2 (56). In addition, high serum levels of IL-1β are a poor prognostic factor in pancreatic cancer patients receiving gemcitabine treatment (57). Accumulating data indicate that IL-1β activates 2 major downstream effectors, the NF-κB and MAPK pathways, to stimulate the expression of adhesion molecules such as ICAM1 and VCAM1 in diverse cell types including tumor cells, thereby amplifying and sustaining responses to IL-1β (54). Through multiplex cytokine array analysis and Western blotting, our data reveal the pivotal role of IL-1β in mediating the interaction between macrophages and HNSCC in inducing ICAM1 expression, thereby enhancing resistance to chemotherapy, particularly DTX. In the present study, we demonstrate a potentially previously unreported mechanism by which IL-1β induces ICAM1 expression via modulation of intracellular ROS levels through SOD2 activation and CAT inhibition in HNSCC. Therefore, IL-1β neutralization or IL-1R blockade in tumor cells represents a direct targeted approach to reducing therapeutic resistance for cancer control. Indeed, our analyses revealed the efficacy of both strategies in decreasing DTX resistance in HNSCC in vitro (Figure 5, I and J). Canakinumab, a human monoclonal antibody against IL-1β, has been established for over a decade in the treatment of rheumatological conditions and other immunological diseases and recently has been shown to be efficacious in reducing lung cancer incidence in a dose-dependent manner (58). However, the clinical efficacy of canakinumab in improving chemotherapeutic efficacy and survival in cancer has yet to be determined.

ATO is a first-line therapeutic agent for APL, which is characterized by a typical chromosomal translocation t(15; 17) (q22; q21) resulting in the formation of the fusion protein, promyelocytic leukemia-retinoic acid receptor α (PML-RARα). ATO directly binds with PML-RARα and enhances product degradation via the ubiquitin-proteasome system, thereby promoting differentiation of APL cells (59). ATO also induces mitochondria-dependent apoptosis of APL cells through inhibition of glutathione peroxidase and the c-Myc–targeted gene, peroxiredoxin III (60). Further, the efficacy of single-agent ATO treatment has been evaluated in a range of solid tumor types including hepatic, esophageal, gastric, pancreatic, ovarian, and prostatic carcinomas (61). Despite promising results both in vitro and in vivo, no significant therapeutic efficacy of ATO has yet been demonstrated in clinical trials (62). Furthermore, compared with APL therapy, higher doses of ATO are required for the treatment of solid tumors, which may cause severe adverse events, such as cardiotoxicity, hepatotoxicity, and nephrotoxicity, thereby limiting the utility of ATO in clinical practice. In an attempt to lower dose-limited toxicity, the use of ATO therapy combined with other chemotherapeutic agents or treatments has emerged as an alternative strategy for treating solid tumors. Accumulating data reveal a synergistic anticancer effect between ATO and cisplatin in the treatment of a diverse range of solid tumors in cell line and animal model studies (63–65). However, clinical outcomes with the use of these combinatorial therapies with ATO have not been as positive as expected, suggesting further research is required to find the optimal combination treatment (62). Recently, the immunoregulatory effects of ATO have been shown to activate T cells through promotion of myeloid-derived suppressor cell differentiation (66) and modulation of macrophage polarization (41), suggesting a regulatory role for ATO in the TME. The results of the present study demonstrate that ATO reduces the infiltration of macrophages into the TME and impairs IL-1β secretion by macrophages, thereby inhibiting ICAM1 activation and leading to the sensitization of HNSCC to DTX. These findings, which were validated by in vivo studies, lay the foundation for use of combinatorial therapy with ATO and DTX as a revolutionary strategy for improving therapeutic efficacy in HNSCC.

## Methods

### Pathological tissue specimens.

The present study protocol was approved by the Ethics Committee of China Medical University Hospital (IRB identifier: CMUH110-REC2-097). A total 54 patients were enrolled in this study from June 2014 to September 2016, with results analyzed retrospectively. Patients provided written informed consent to inclusion in the present study. Demographic characteristics of patients with HNSCC are shown in Table 1. The chemotherapy regimen was the same as used in our previous study (9). Therapeutic response to TPF ICT was evaluated according to the RECIST 1.1 (22). In the present study, we defined positive and negative responses as ≥70% and <70% decreases in the sum of the longest diameter of the target lesions compared with baseline, respectively. Pathological tissue specimens acquired from the oral cavity, oropharynx, and hypopharynx sections were used for IHC analyses of progression-related protein markers.

### Cell lines and cell culture.

The human pharyngeal squamous cell carcinoma cell line, FaDu, was purchased from the ATCC. The human oral squamous carcinoma cell line, OECM-1, was purchased from MilliporeSigma. The human tongue squamous cell carcinoma cell line, SCC-25, and the esophageal carcinoma cell line, CE 146T*VGH* (CE146T), were obtained from the Bioresource Collection and Research Center, Taiwan. The human tongue squamous cell carcinoma cell line, SAS, was obtained from Japanese Collection Research Bioresources. FaDu and CE146T cells were maintained in DMEM (Invitrogen). SAS cells were maintained in DMEM supplemented with 2 mM l-glutamine. OECM-1 cells were maintained in RPMI 1640 media (Invitrogen) supplemented with 2 mM l-glutamine. SCC-25 cells were cultured in DMEM/F-12 media (Invitrogen) supplemented with 0.5 mM sodium pyruvate and 400 ng/mL hydrocortisone. The human leukemia monocytic cell line, THP-1, was obtained from ATCC and grown in suspension in RPMI/GlutaMAX (Invitrogen) supplemented with 10% FBS. All cell lines were grown in a humidified atmosphere of 5% CO_2_ and 95% air at 37°C.

### Chemical reagents.

PMA (catalog 10008014) was purchased from Cayman Chemical. NAC (catalog A7250) and cis-diammineplatinum(II) dichloride (catalog 479306) were purchased from MilliporeSigma. MTT (catalog M6494), DCF (catalog C6827), and MitoSOX (catalog M36008) were purchased from Invitrogen. The ICAM1 inhibitor, A205804 (catalog sc-203484), was purchased from Santa Cruz Biotechnology. The IL-1R antagonist, anakinra (catalog HY-108841), was purchased from MedChemExpress. GM-CSF and M-CSF were purchased from PeproTech. GRO-α, IL-1α, IL-1β, IL-18, MIP-1β, SDS-1α, and VEGF were purchased from CROYEZ. Neutralizing antibodies against human IL-1β (4H5) and isotype-matched IgG (T8E5) were purchased from InvivoGen. ELISA kits for human IL-1β (catalog ARG80101) and mouse IL-1β (catalog ARG80196) were purchased from Arigo Biolaboratories. Clinical medications used in the present study and corresponding suppliers were DTX (Enzo), axitinib (Pfizer), PIO (Takeda), atezolizumab (Roche), cabozantinib (Ipsen), and Asadin (TTY Biopharm).

### In silica analyses.

Pairwise gene expression correlation analyses were performed on the GEPIA web server (http://gepia.cancer-pku.cn/) using TCGA and GTEx expression data by a standard processing pipeline. The monotonic relationship between ICAM1 and CD163 expression was calculated using Spearman’s correlation coefficient.

### Induction of monocyte differentiation.

Primary PBMCs from the sera of healthy donors, our team members, and THP-1 cells were seeded onto Transwell inserts (24 mm diameter, 0.4 μm pore size; Corning) at a density of 3 × 10^6^ cells/insert and 1 × 10^6^ cells/insert, respectively. Monocyte differentiation into macrophages was induced by treatment with 100 ng/mL PMA for 48 hours followed by culture in complete medium (RPMI/10% FBS and 1% antibiotics) without PMA for 24 hours (67). FaDu or OECM-1 cells were seeded in 6-well plates at a density of 5 × 10^5^ cells/well and allowed to attach overnight. For coculture, both macrophages and FaDu or OECM-1 cells were merged into 1 well and cocultured in a humidified atmosphere of 5% CO_2_ and 95% air at 37°C for 24 hours. After centrifugation, coculture conditional media were aliquoted and stored at –30°C until further use. A 10-fold concentration of coculture conditional media was prepared by centrifugation at 4,000*g* at 4°C for 1 hour using 3K Macrosep advanced centrifugal devices (Pall life sciences) with the final 20% and 40% preparations of concentrated CM designated CM 2× and CM 4×, respectively.

### Mitochondrial superoxide and intracellular ROS measurements.

For flow cytometry measurements, trypsinized tumor cells (3 × 10^5^ cells) were treated with 10 μM DCF at 37°C for 30 minutes or 5 μM MitoSOX at 37°C for 10 minutes and then analyzed using a BD FACSCalibur flow cytometer system and CellQuest software. For fluorescent image analyses, tumor cells (1 × 10^5^ cells) were seeded onto 12 mm glass coverslips in 6-well plates overnight and then treated with IL-1β for a further 24 hours. Tumor cells stained with MitoSOX were detected using a Leica TCS SP8 X confocal spectral microscope imaging system. Tumor cells stained with DCF were detected using a fluorescence microscope (ZEISS AX10).

### Transwell migration and invasion assays.

For in vitro migration assays, HNSCC cells (0.5 × 10^5^ cells in 200 μL) were suspended in the upper half of a PET membrane Transwell insert chamber (BD Biosciences) on a 24-well plate. For in vitro invasion assays, tumor cells (1.5 × 10^5^ cells in 200 μL) were suspended in Transwell insert chambers coated with Matrigel (1 mg/mL; BD Biosciences). Media without FBS supplementation were added into the upper chamber, whereas media with 10% FBS supplementation were added into the lower chamber. After incubation at 37°C for 24 hours or 48 hours for migration and invasion assays, respectively, tumor cells that had passed through the insert were fixed with 3.7% formalin (MilliporeSigma) and stained with 0.1% crystal violet (MilliporeSigma). For quantification, crystal violet was extracted using 50% ethanol and 0.1% acetic acid and subjected to colorimetric measurement at 570 nm.

### Cell viability assays.

The effects of chemotherapeutic drugs and CM on cell viability were determined using the MTT method. Tumor cells were seeded into 24-well microplates at a density of 2 × 10^4^ cells/well and treated with various reagents at the designed doses of chemotherapeutic drugs, such as DTX, for 48 hours. After treatment, 200 μL MTT solution (1 mg/mL in PBS) was added for 4 hours at 37°C. After removing the solution, 500 μL DMSO was used to dissolve insoluble purple formazan dyes. Cell viability was calculated by the optical density (OD) at the wavelength of 570 nm. The viability rate was defined as cell viability (%) = (experiment OD_570_/control OD_570_) × 100%.

### Proteomic identification.

Proteomic alterations in FaDu and OECM-1 cells induced by macrophage coculture were identified by mass spectrometric (MS) analysis. Total proteins were extracted from FaDu and OECM-1 cells using RIPA lysis and extraction buffer (Thermo Fisher Scientific) and sonication. Protein concentrations were determined using Bio-Rad Protein Assay kits by measuring absorbance at 595 nm. Total protein samples (40 μg) were separated using 10% SDS-PAGE and divided into 5 gel fractions. After fine cutting (<1 mm^3^), gel pieces were subjected to in-gel digestion to produce tryptic peptides. An Orbitrap Fusion mass spectrometer (Thermo Fisher Scientific) equipped with the Ultimate 3000 RSLC system (Dionex) and a nano-electrospray ion source (New Objective) was used for MS analysis. The survey scan was set at a mass range (*m*/*z*) of 375–1,500 (automatic gain control target, 4 × 10^5^) and resolution of 120,000 at *m*/*z* 200. The 20 most abundant multiple-charged ions were sequentially fragmented by collision-induced dissociation for tandem mass analysis. Protein identification and label-free quantification were performed using the computational platform, Proteome Discovery (v2.4). The identification threshold was set at a *P* < 0.05.

### Western blotting.

Protein expression levels were determined by 9.5% or 13% SDS-PAGE separation and Western blot assays dependent on the molecular weight of target proteins. For Western blotting, proteins were transferred onto PVDF membranes at 400 mA at 0°C for 3 hours in 25 mM Tris-HCl, 197 mM glycine, and 13.3% (v/v) methanol. Membranes were blocked with 5% (*w/v*) skim milk in TBS with 0.1% Tween 20 (TBST) for 1 hour and then incubated with primary antibodies at 4°C for 16 hours. The list of primary antibodies used in the present study is in Supplemental Table 5. After membranes were washed for 15 minutes in TBST 3 times, horseradish peroxidase–conjugated secondary antibodies were added, and membranes were incubated at room temperature for 1 hour. After the same washing procedure, immunoreactive signals were revealed using an enhanced ECL substrate Western Lighting Plus-ECL (PerkinElmer) and recorded by developing photographic film under optimum exposure conditions.

### Quantitative polymerase chain reaction.

Total RNA was extracted from HNSCC cells with or without CM treatment using TRIzol reagent (Invitrogen). Total RNA was used for reverse transcription PCR (RT-PCR) using MMLV first-strand synthesis kits (GeneDireX). Diluted RT-PCR products were applied for quantitative PCR (qPCR) analysis using SYBR FAST qPCR Master Mix Kits (Kapa Biosystems) using the LightCycler 480 apparatus (Roche). GAPDH, β-actin, and 18S rRNA were used as endogenous controls. The sequences of qPCR primers used in the present study are summarized in Supplemental Table 6. The mRNA expression levels were determined by the comparative Ct method using 2^−ΔΔCt^.

### Cytokine measurements.

Cytokine levels in CM were measured using the Human Immune Monitoring 65-Plex ProcartaPlex Panel (Thermo Fisher Scientific, catalog EPX650-16500-901), which is used to analyze 65 cytokine targets in a single well by Luminex xMAP technology. A total of 50 μL of cell culture supernatant was incubated with Antibody Magnetic Beads and Detection Antibodies according to the manufacturer’s assay protocol. Quantitative data were acquired on a Luminex 200 analytic instrument. Data analyses were performed using the supporting analysis software.

### Mouse model of orthotopic HNSCC and antitumor assays.

The animal procedure (CMUIACUC-2021-161) of the present study was approved by the Institutional Animal Care and Use Committee at China Medical University Hospital. Orthotopic mouse models of HNSCC were established by injecting OECM-1 or SAS cells into the tongues of 5-week-old male BALB/c nude mice (BALB/cAnN.Cg-*Foxn1^nu^*/CrlNarl) or NOD/SCID mice (BioLASCO Taiwan Co., Ltd). Mice were anesthetized with 25 mg/kg of Zoletil 50 and 10 mg/kg of Rompun by intraperitoneal injection. A total of 1 × 10^6^ OECM-1 cells (SAS cells; results shown in Supplemental Figure 8) in 20 μL serum-free DMEM were inoculated by syringe with a 31 gauge needle at the lateral border of the tongue. Tongue tumors were established 6 days after injection. Mice were randomized and assigned to 4 groups for treatment: (i) the control group (saline treatment, *n* = 5); (ii) ATO group (*n* = 5); (iii) DTX group (*n* = 5); and (iv) ATO+DTX group (*n* = 5). Mice receiving 0.5 mg/kg ATO and/or 1 mg/kg DTX were subjected to intraperitoneal injection on 5 consecutive days with 2 rest days for 2 cycles. Animals were sacrificed on day 18, and tumors were excised for IL-1β measurements or fixation in formalin for IHC analyses. At day 6 and day 18, tumor volumes were estimated using the following formula: tumor volume = (length × width × height)/2, where length represents the largest tumor diameter, width represents the perpendicular tumor diameter, and height represents tumor thickness.

### Immunohistochemical assay.

Immunohistochemical assay was performed using an automatic BenchMark XT staining machine (Ventana Medical Systems) and iView DAB detection kits (Ventana Medical Systems). Paraffin sections (4 μm) containing human HNSCC tissue specimens were deparaffinized, hydrated, and heated to 95°C–100°C for 4 minutes for antigen retrieval. After inactivating endogenous peroxidase activity, rabbit anti–human CD163 monoclonal antibody (Cell Signaling Technology 93498; 1:500 dilution), rabbit anti–human ICAM1 polyclonal antibody (Cell Signaling Technology 4915; 1:50 dilution), rabbit anti–human CD44 recombinant antibody (Abcam ab51037), and rabbit anti–mouse F4/80 antibody (Abcam ab2228115) were used for immunohistochemical staining. Tissue sections were incubated with iView copper for 4 minutes to enhance signal intensity. Tissue specimens were counterstained with hematoxylin, dehydrated, mounted, and observed using an Eclipse E600 light microscope (Nikon). All immunohistochemical analyses were evaluated by an experienced histologist.

### Statistics.

Data were displayed as the means ± SD. The significance of differences was examined by 2-tailed Student’s *t* test or 1-way ANOVA followed by Tukey’s post hoc test. OS and PFS were determined by the Kaplan-Meier method. Survival curves were compared using the log-rank test. Statistical analyses were performed using IBM SPSS Statistics 22. *P* < 0.05 was considered statistically significant.

### Study approval.

The present study in humans was approved by the Ethics Committee of China Medical University Hospital (IRB identifier: CMUH110-REC2-097), Taichung, Taiwan. Patients provided written informed consent prior to participation in this study. The present study in animals was approved by the Institutional Animal Care and Use Committee at China Medical University Hospital (identifier: CMUIACUC-2021-161), Taichung, Taiwan.

## Author contributions

CYH, CCL, YWH, JHC, YAT, LCC, CCF, and WCC performed experiments. CYH, CCL, CYL, and WCC advised on most of the experiments. CYH, CCL, LCC, CYL, and WCC designed experiments, analyzed data, and wrote the manuscript. All authors discussed results and commented on the manuscript.

## Supplementary Material

Supplemental data

Supplemental table 5

## Figures and Tables

**Table 1:**
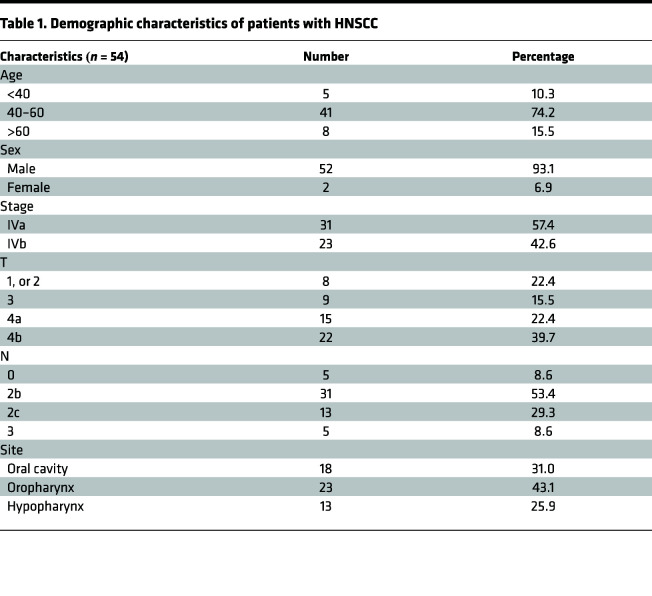
Demographic characteristics of patients with HNSCC

## Version 1 10/20/2022

In-Press Preview

## Version 2 12/08/2022

Electronic publication
