# Supplementary material for: Macrophage secretory IL-1β promotes docetaxel resistance in head and neck squamous carcinoma via SOD2/CAT-ICAM1 signaling
Source: JCI Insight. 2022 Dec 8;7(23):e157285. doi: 10.1172/jci.insight.157285 (PMC9746909; doi:10.1172/jci.insight.157285)
Supplement: Supplemental data [file jciinsight-7-157285-s175.pdf]

Macrophage secretory IL-1β promotes docetaxel resistance in head and neck squamous carcinoma via SOD2/CAT-ICAM1 signaling

Supplementary Information

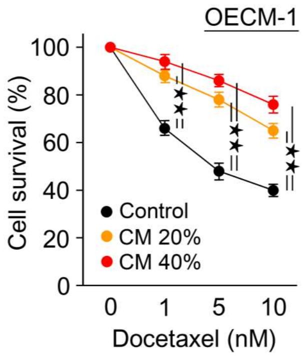

Supplementary Figure 1. CM enhances resistance of OECM-1 to docetaxel.

OECM-1 cells were pretreated with different doses of CM for 24 hr and then treated with indicated doses of DTX for another 48 hr. Cell viability was determined by MTT assay.

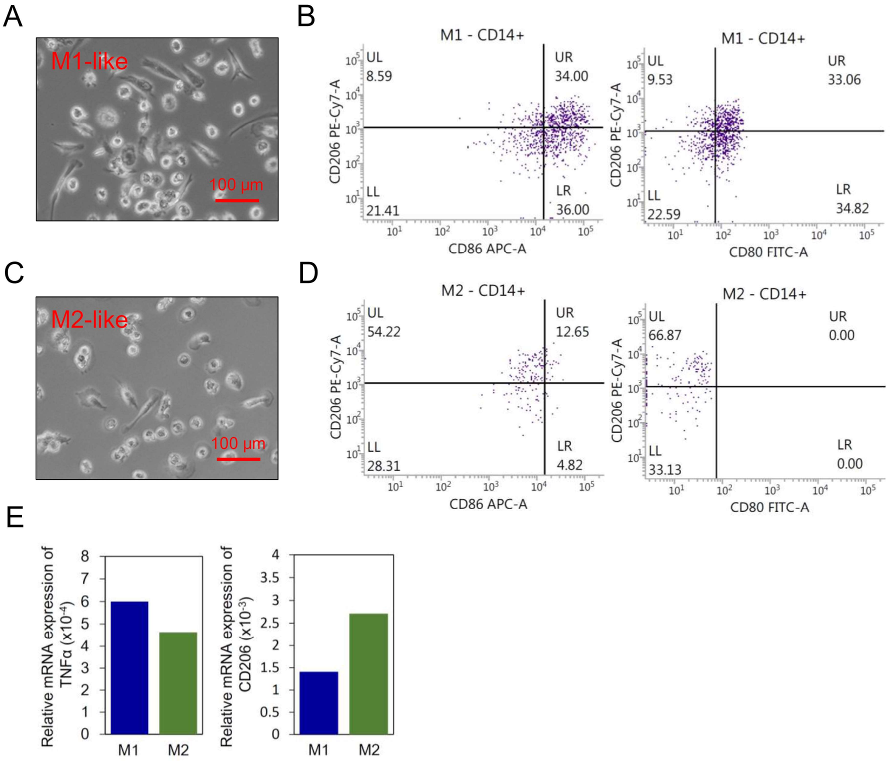

Supplementary Figure 2. Characterization of PBMC-derived macrophages.

M1 polarization was achieved by supplementation with interferon-γ (10 ng/mL) and lipopolysaccharides (100 ng/mL) for 48 hr, whereas M2 polarization was obtained by supplementing cells with interleukin-4 (20 ng/mL) for 48 hr. After polarization, (**A**) M1 macrophages showed a spindle shaped morphology, while (**C**) M2 macrophages had a more spread morphology. Flow cytometry assay revealed that (**B**) M1 macrophages showed $CD80^{+}$ $CD86^{+}$ $CD206^{low}$, while (**D**) M2 macrophages showed $CD80^{-}$ $CD86^{-}$ $CD206^{high}$ profile. (**E**) The results of gene expression assays performed by RT-qPCR confirm the polarization of M1 and M2, M1 macrophages showed relatively higher TNF-α and lower CD206 expression levels in comparison with M2 macrophages.

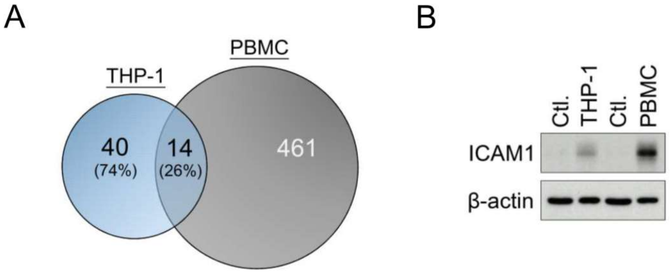

Protein Name	Gene Name
Intercellular adhesion molecule 1	ICAM1
Laminin subunit gamma-2	LAMC2
Interferon-stimulated gene 20 kDa protein	ISG20
Interleukin-1 alpha	IL1A
Matrix metalloproteinase-14	MMP14
EKC/KEOPS complex subunit TP53RK	TP53RK
Prostaglandin G/H synthase 2	PTGS2
Fibronectin type-III domain-containing protein 3A	FNDC3A
FK506-binding protein 15	FKBP15
OTU domain-containing protein 4	OTUD4
Interferon-induced protein with tetratricopeptide repeats 3	IFIT3
Laminin subunit beta-3	LAMB3
Superoxide dismutase [Mn], mitochondrial	SOD2
Interferon-induced protein with tetratricopeptide repeats 1	IFIT1

Supplementary Figure 3. CM induces ICAM1 in FaDu cells.

(**A**) The proteomic alterations of 5-fold up-expression in FaDu cells induced by THP-1-derived and PBMC-derived macrophages were displayed by the venny analysis. The commonly induced proteins were listed below. (**B**) ICAM1 expression in FaDu cells cocultured with by THP-1-derived and PBMC-derived macrophages was determined by western blot assay.

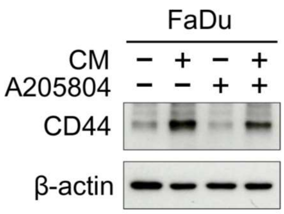

Supplementary Figure 4. ICAM1 inhibitor A205804 attenuates CM-induced CD44 in FaDu cells.

The expression of CD44 in FaDu cells with or without 20% CM treatment in the presence or absence of 10 μM ICAM1 inhibitor was determined by western blot assay. β-actin, loading control.

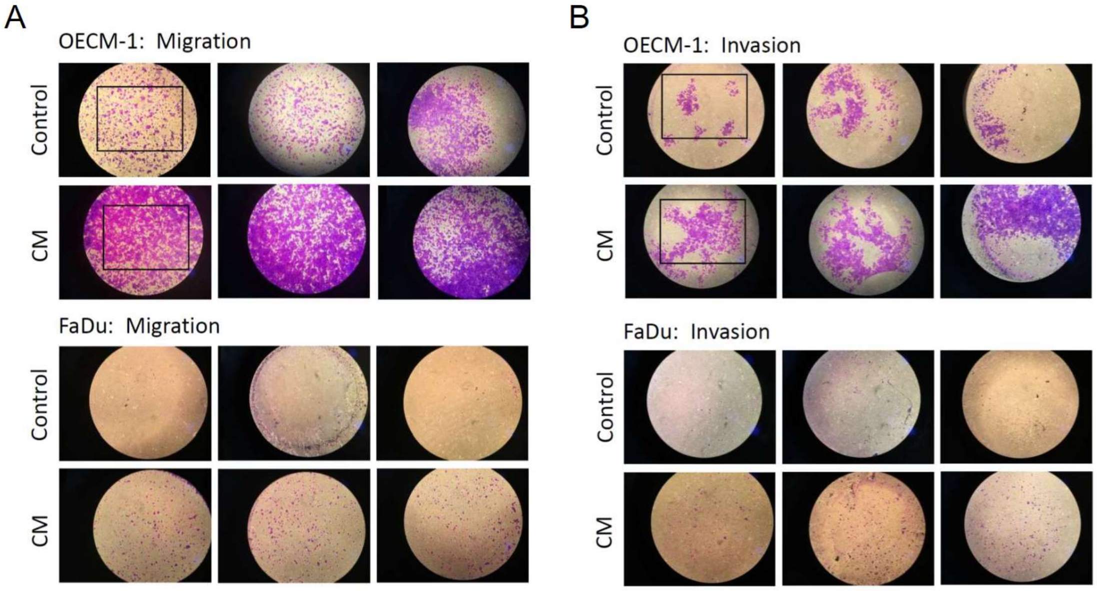

Supplementary Figure 5. CM increase the abilities of migration and invasion in HNSCC cells.

(**A**) Transwell migration assay and (**B**) transwell invasion assay were performed by OECM-1 and FaDu cells with or without 10% CM treatment. The mark areas serve as the represented images shown in Figure 2N and 2O in the text.

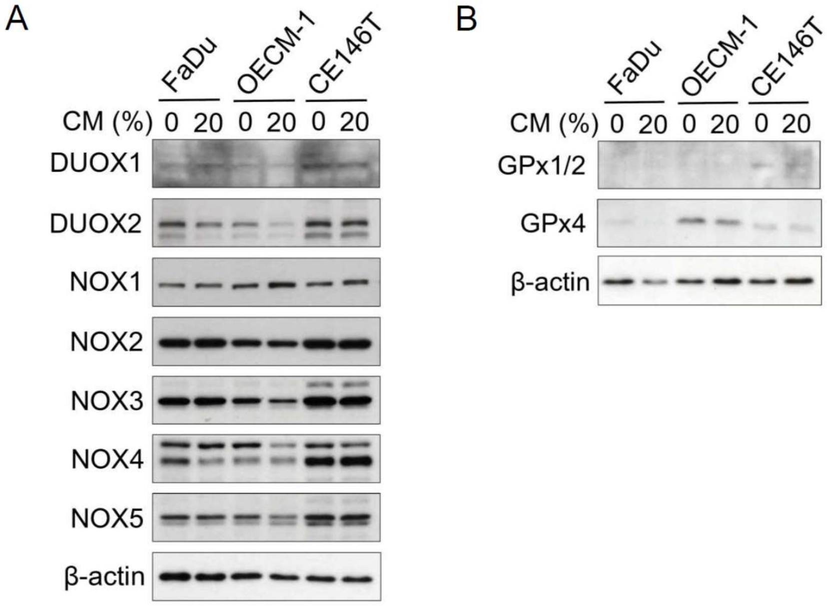

Supplementary Figure 6. CM has no effect on the expression of NOXs and GPxs in HNSCC cells.

FaDu, OECM-1, and CE146T cells were treated with 20% CM for 24 hr, the expression of (**A**) NOX proteins and (**B**) GPx proteins were determined by western blot assay. β-actin serves as the loading control.

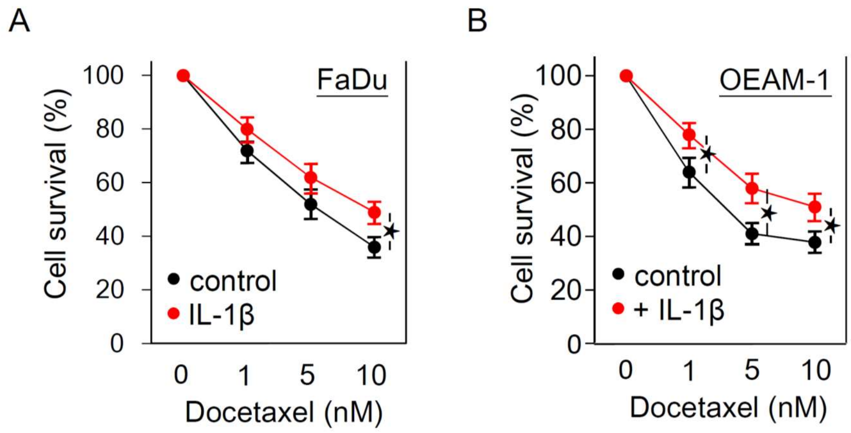

Supplementary Figure 7. IL-1β increases resistance of HNSCC to docetaxel.

(**A**) FaDu and (**B**) OECM-1 cells were pretreated with IL-1β (3 ng/mL) for 24 hr, and then treated with indicated doses of DTX for another 48 hr. Cell viability was determined by MTT assay.

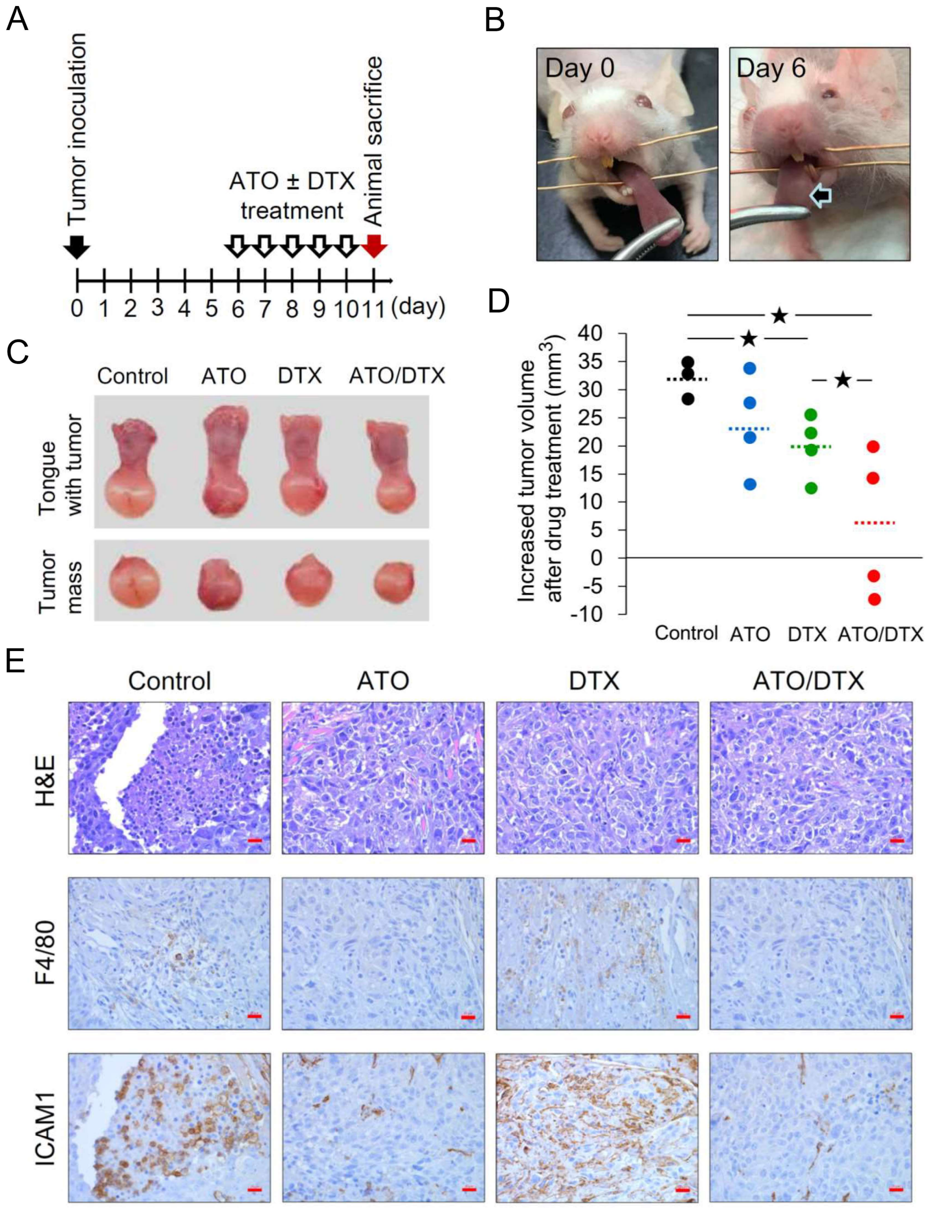

Supplementary Figure 8. The combination of ATO improves efficacy of DTX to SAS cells in preclinical mouse model.

(**A**) Schematic illustration of the animal experiment. (**B**) The representative images of the tongue before tumor cell inoculation (Day 0) and the visible tumor mass (indicated by arrow) in the tongue (Day 6). (**C**) The animals were sacrificed at the 11th day, the representative images showed tumor masses in the tongues of mice subjected with differential drug treatments. (**D**) Mice were randomized assigned into four groups for treatment, including (1) the control group (saline treatment, *N*=3); (2) ATO group (*N*=4); (3) DTX group (*N*=4); (4) ATO+DTX group (*N*=4). Increased tumor volume of individual mouse after drug treatment (from the 6th day to the 11th day) was calculated and plotted. Dash lines showed the average increased tumor volume in each group. *,

<0.05; ★★, <0.01. (**E**) Tumor masses were sectioned and embedded in paraffin. IHC analysis was performed with the indicated antibodies. Scale bar, 20 μm.
